# Dimethyl sulfoxide stimulates the AhR-Jdp2 axis to control ROS accumulation in mouse embryonic fibroblasts

**DOI:** 10.1007/s10565-021-09592-2

**Published:** 2021-03-15

**Authors:** Kenly Wuputra, Ming-Ho Tsai, Kohsuke Kato, Ya-han Yang, Jia-Bin Pan, Chia-Chen Ku, Michiya Noguchi, Shotaro Kishikawa, Koji Nakade, Hua-Ling Chen, Chung-Jung Liu, Yukio Nakamura, Kung-Kai Kuo, Ying-Chu Lin, Te-Fu Chan, Deng-Chyang Wu, Ming-Feng Hou, Shau-Ku Huang, Chang-Shen Lin, Kazunari K. Yokoyama

**Affiliations:** 1 Graduate Institute of Medicine, Kaohsiung Medical University, Kaohsiung, Taiwan; 2 Regenerative Medicine and Cell Therapy Research Center, Kaohsiung Medical University, Kaohsiung, Taiwan; 3 School of Medicine, Kaohsiung Medical University, Kaohsiung, Taiwan; 4 Department of Infection Biology, Graduate School of Comprehensive Human Sciences, University of Tsukuba, Tsukuba, Japan; 5 Cell Therapy and Research Center, Kaohsiung Medical University Hospital, Kaohsiung, Taiwan; 6 Cell Engineering Division, RIKEN BioResource Research Center, Tsukuba, Japan; 7 Gene Engineering Division, RIKEN BioResource Research Center, Tsukuba, Ibaraki, Japan; 8 National Institute of Environmental Health, National Health Research Institutes, Zhunan, Taiwan; 9 Department of Gastroenterology, Kaohsiung Medical University Hospital, Kaohsiung, Taiwan; 10 Department of Surgery, Kaohsiung Medical University Hospital, Kaohsiung, Taiwan; 11 School of Dentistry, Kaohsiung Medical University, Kaohsiung, Taiwan; 12 Department of Obstetrics and Gynecology, Kaohsiung Medical University Hospital, Kaohsiung, Taiwan; 13 Department of Biological Sciences, National Sun Yat-sen University, Kaohsiung, Taiwan

**Keywords:** AhR, Dimethyl sulfoxide, Jun dimerization protein 2, Nrf2, Mouse embryonic fibroblasts, Reactive oxygen species

## Abstract

The aryl hydrocarbon receptor (AhR) is a ligand-binding protein that responds to environmental aromatic hydrocarbons and stimulates the transcription of downstream phase I enzyme–related genes by binding the *cis* element of dioxin-responsive elements (DREs)/xenobiotic-responsive elements. Dimethyl sulfoxide (DMSO) is a well-known organic solvent that is often used to dissolve phase I reagents in toxicology and oxidative stress research experiments. In the current study, we discovered that 0.1% DMSO significantly induced the activation of the AhR promoter via DREs and produced reactive oxygen species, which induced apoptosis in mouse embryonic fibroblasts (MEFs). Moreover, Jun dimerization protein 2 (Jdp2) was found to be required for activation of the AhR promoter in response to DMSO. Coimmunoprecipitation and chromatin immunoprecipitation studies demonstrated that the phase I–dependent transcription factors, AhR and the AhR nuclear translocator, and phase II–dependent transcription factors such as nuclear factor (erythroid-derived 2)–like 2 (Nrf2) integrated into DRE sites together with Jdp2 to form an activation complex to increase AhR promoter activity in response to DMSO in MEFs. Our findings provide evidence for the functional role of Jdp2 in controlling the AhR gene via Nrf2 and provide insights into how Jdp2 contributes to the regulation of ROS production and the cell spreading and apoptosis produced by the ligand DMSO in MEFs.

**Graphical abstract:**

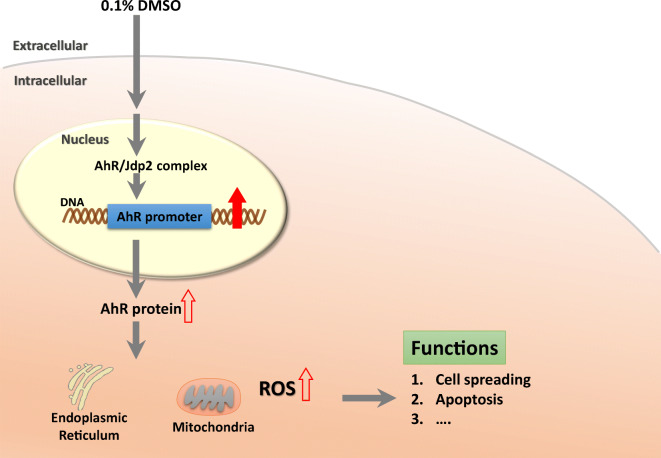

**Supplementary Information:**

The online version contains supplementary material available at 10.1007/s10565-021-09592-2.

## Introduction

Dimethyl sulfoxide (DMSO) is an amphiphilic organic reagent used in biomedical research for various kinds of experiments (Capriotti and Capriotti 2012). DMSO displays various characteristics, such as anti-inflammatory, diuretic, vasodilatory, and bacteriostatic activities (Rivers-Auty and Ashton 2013). In experiments in vitro, it is used for cryopreservation and is a solvent recommended for dissolving small hydrophobic reagents and drugs, including toxins (Capriotti and Capriotti 2012; Rivers-Auty and Ashton 2013). DMSO interacts with phospholipids, which facilitate the passage of drugs across membranes (Notman et al. 2007), and is a scavenger of free radicals at low doses but become prooxidative at higher doses (Sanmartin-Suarez et al. 2011). DMSO remains a solvent of choice for biomedical experiments, despite its potential cellular interference and cytotoxic effects (da Silva et al. 2004; Laskar et al. 2010; Sadowska-Bartosz et al. 2013; Rawls et al. 2017). Thus, it remains critical to determine the possible interactions of this solvent with various biological systems and to establish the optimal, nontoxic concentration of DMSO, and the specific experimental conditions, to ensure the fidelity of experimental results (Capriotti and Capriotti 2012; da Silva et al. 2004; Laskar et al. 2010; Sadowska-Bartosz et al. 2013; Rawls et al. 2017).

The aryl hydrocarbon receptor (AhR) plays a role in the transcription of target genes that are activated by xenobiotics, environmental disruptors, bacteria, and inflammatory substances (Tarnow et al. 2019). After binding to the phase I ligands, the AhR moves into the nucleus via dimerization with the AhR nuclear translocator (Arnt). Subsequently, the AhR-Arnt complex binds to the dioxin-responsive elements (DREs)/xenobiotic-responsive elements with a minimum core sequence of 5′–GCGTG–3′ in the genes that encode phase I enzymes, such as the cytochrome P450 family 1 subfamily A member 1 (Cyp1a1), Cyp1a2, and Cyp1b1 (Lo and Matthews 2012; Miao et al. 2005; Swanson et al. 1995; Whitlock Jr. 1999; Zhang et al. 1998). The AhR- and Arnt-binding sites overlap (Lo and Matthews 2012), regardless of the ligand binding. The expression of genes for phase II enzymes largely relies on nuclear factor (erythroid-derived 2)–like 2 (Nrf2), which recognizes antioxidant-response elements (AREs; 5′–G/ATGACNNNGC–3′) on the gene promoters (Friling et al. 1990; Primiano et al. 1997). Nrf2 is the “master controller” of the cellular antioxidative response. Upon exposure to phase II ligands, dissociation of Nrf2 from the Kelch-like ECH-associated protein 1 (Keap1) leads to the shuttling of Nrf2 into the nucleus, where it binds to small Maf protein family members (e.g., MafK, MafF, and MafG), to form an active complex for transcriptional activation, and induces ARE-dependent responses (Blank 2008; Katsuoka et al. 2005; Kwak et al. 2004; Yamamoto et al. 2018).

The AhR ligands include dioxins, flavonoids, and polycyclic aromatic hydrocarbons, which induce the cytochrome P450 family to mediate production of reactive oxygen species (ROS) and electrophilic metabolites (Klotz and Steinbrenner 2017; Rushmore and Kong 2002). This process induces the antioxidative response by activating Nrf2- and ARE-dependent phase II enzymes, such as glutathione S-transferases, which help maintain ROS homeostasis (Katsuoka et al. 2005; Moldogazieva et al. 2018; Wolfle et al. 2014; Fuyuno et al. 2018). AhR and Nrf2 also coordinate the expression of other phase II enzymes, such as NAD(P)H dehydrogenase 1 (quinone 1) (Nqo1), UDP-glucuronosyltransferase family 1 member A1 (Ugt1A1), and heme oxygenase 1 (Ho1) (Fuyuno et al. 2018; Kohle and Bock 2007; Wang et al. 2013). These target genes, known as the “AhR-Nrf2 gene battery,” are regulated by crosstalk between AhR- and Nrf2-dependent signaling (Haarmann-Stemmann et al. 2012; Tsuji et al. 2012; Yeager et al. 2009). However, the molecular interactions between AhR and Nrf2 signaling networks that underlie the precise regulation of this gene battery remain poorly understood.

Mouse embryonic fibroblasts (MEFs) prepared from embryos on gestation days 14.5 to 15.5 form a spindle shape when cultured in vitro and display typical fibroblast features. Experimentally, they are used as feeder layers and as a substrate to maintain the pluripotency, enhance the plating efficiency, and support the growth and survival of embryonic stem cells (Lin and Talbot 2011). In 1963, MEFs were immortalized by repeated transmission to produce the NIH 3T3 cell line (Todaro and Green 1963). MEFs have also been used as an embryonic model to better understand the mechanisms of action of specific phase I toxicants, such as fumonisin B1 (Flynn et al. 1997), cadmium (He et al. 2008), and 2,3,7,8-tetrachlorodibenzo-p-dioxin (TCDD) (Birnbaum and Harris 1991). Furthermore, MEFs can be used to evaluate gene functions in gene depletion studies, and MEFs can be collected from knockout (KO) mice in which the gene depletion results in no viable offspring (Beg et al. 1995). More recently, MEFs have been widely used in stem cell biology, after MEFs were reprogrammed into induced pluripotent stem cells (Takahashi and Yamanaka 2006).

The Jun dimerization protein 2 (Jdp2), which is a member of the activator protein 1 family, participates in positive and negative transcriptional regulation and control of chromatin assembly (Aronheim et al. 1997; Jin et al. 2002; Tsai et al. 2016). Previously, we determined that Jdp2 binds directly to the ARE and to the Nrf2-MafK complex, to contribute to the activation of the ARE-dependent antioxidative response (Chiou et al. 2013; Tanigawa et al. 2013).

Here, we report that Jdp2 also regulates AhR expression at the transcriptional level in response to DMSO, indicating that the DMSO-induced activation of the *AhR* promoter is regulated by the interaction of Jdp2, Nrf2, and AhR. Given that Jdp2 also positively regulates ARE-dependent phase II gene promoters (Chiou et al. 2013; Tanigawa et al. 2013), this protein likely serves as a bifunctional activator to link the AhR and Nrf2 responses, which contribute to the maintenance of ROS homeostasis and support cell apoptosis in response to DMSO.

## Materials and methods

### Animals, cell culture, and reagents

All experiments were conducted in accordance with the approved guidelines for animal welfare and the care of laboratory animals issued by the Animal Care Committee of RIKEN Bioresource Research Center (BRC) in Japan, and the National Laboratory Animal Center, and Kaohsiung Medical University in Taiwan. The strategy used to generate *Jdp2*^−/−^ mice was as described elsewhere (Nakade et al. 2007; Pan et al. 2010). Primary MEFs were cultivated in Dulbecco’s Modified Eagle’s Medium (Hyclone, high glucose; GE Healthcare, Pittsburgh, PA, USA) including 10% fetal bovine serum (FBS; Invitrogen, Grand Island, NY, USA), as described previously (Nakade et al. 2007; Pan et al. 2010). Normal, mouse NIH3T3 fibroblasts, human hepatoblastoma HepG2 cells, human diploid lung fibroblast WI38 cells, and human 293T cells were purchased from the RIKEN BRC (Tsukuba, Japan). DMSO was obtained from Sigma-Aldrich (St. Louis, MO, USA).

### Viral vector construction and antibodies

*pcDNA-Nrf2*, *pcDNA-MafK*, and *pcDNA-Jdp2* were obtained from RIKEN BRC (Tanigawa et al. 2013). *pQCXIN-CA-AhR-EGFP* was a gift from Dr. YF Kuriyama, Tsukuba University (Tsukuba, Japan). DRE-luciferase was a gift from Dr. Y H Cheng, (China Medical University, Taiwan; Cheng et al. 2008), and ARE-luciferase (*pGL4-hQR25*-firefly luciferase) was as described previously (Tanigawa et al. 2013). All antibodies used in this study are described in Supplementary Table S1.

### Transfection and luciferase reporter assay

MEFs in 24-well plates (4 × 10^5^ cells) were cultivated for 24 h and then cotransfected with 500 ng of AhR-luciferase plasmid and 10 ng of pRL-CMV plasmid (encoding *Renilla* luciferase) using Lipofectamine 2000 (Invitrogen) or polyethylenimine (PEI; linear, MW25,000, cat. no. 23966; Polysciences, Warrington, PA, USA). For forced expression studies, cells were cotransfected with pcDNA3 encoding Nrf2, MafK, AhR, or Jdp2. The amount of all transfected DNA was prepared at 1 μg/well via the addition of a pBluescript plasmid. The transfected cells were treated with DMSO for the indicated time and harvested 48 h after transfection. Luciferase activity was determined on a GloMax20/20 Luminometer using a dual-luciferase reporter assay following the manufacturer’s instructions (Promega, Madison, WA, USA). The reporter activity was calculated as the relative luciferase activity (*firefly* luciferase/*Renilla* luciferase) and expressed as fold induction over that of the empty vector in wild-type (WT) MEFs. All measurements were conducted in triplicate, and values are shown as means ± standard error of the mean (SEM) from three to five independent experiments.

### Immunocytochemistry

Formaldehyde (4%) was added to fix MEFs for 10 min and then washed with phosphate-buffered saline (PBS). To block nonspecific binding, the MEFs were incubated in a blocking solution included 10% FBS and 0.1% Triton X-100 in PBS for 15 min, followed by overnight incubation with the primary antibodies. After washing with PBS-T, the cells were treated for 90 min with the following secondary antibodies, Alexa Fluor 594-labeled goat anti-rabbit immunoglobulin G (IgG, A11037; Life Technologies, Thermo Fisher Scientific; Waltham, MA, USA), Alexa Fluor 594-labeled goat anti-mouse IgG (A11032; Life Technologies, Thermo Fisher Scientific), and Alexa Fluor 488-labeled goat anti-rabbit IgG (A11034; Life Technologies, Thermo Fisher Scientific), followed by processing using 4′,6′-diamino-2-phenylindole (DAPI), to detect cell nuclei (1:3000; 5 mg/mL stock in DMSO; Merck, Darmstadt, Germany). Cells were mounted on slides using ProLong Gold Antifade mounting medium (Molecular Probes, P36034; Thermo Fisher Scientific), and cell immunofluorescence was detected using an Olympus FV1000 confocal laser scanning microscope (Olympus, Tokyo, Japan).

### SDS-polyacrylamide gel electrophoresis (SDS-PAGE), immunoprecipitation, and western blotting

SDS-PAGE, immunoprecipitation, and western blot assays were carried out as described previously (Tanigawa et al. 2013; Pan et al. 2010). In brief, cell lysates were centrifuged at 500 × *g* for 5 min at 4 °C to prepare nuclei and cytosol by using the nuclear and cytoplasmic extraction reagents, respectively (NE-PER; 78833; Thermo Fisher Scientific). Cell lysates were fractionated in 10% SDS-PAGE and transferred to Immobilon-P polyvinylidene difluoride (PVDF) membranes (0.45-μm IPVH00010; Merck) for 1 h at 100 V (fixed) at 10°C using a Mini Trans-Blot transfer system (Bio-Rad Laboratories, Hercules, CA, USA). Blotted proteins were visualized with Ponceau S (Merck, P17170) to examine the amounts of the transferred proteins. PVDF membranes were then incubated with primary and secondary antibodies (Supplementary Table S1). Results were investigated using a ChemiDoc XRS Plus instrument (Bio-Rad). Immunoprecipitation was conducted by protein A/G beads coated by indicated antibodies (Tanigawa et al. 2013; Pan et al. 2010).

### Chromatin immunoprecipitation

MEFs were collected using a plastic policeman and fixed in 1% formaldehyde in PBS for 8 min at room temperature. Glycine (0.125 M) was added to the solution and then incubated at room temperature for 5 min, to quench the cross-linked products. Cells were harvested using cold PBS with protease inhibitors and washed three times each at 4 °C, with 5 min of rotation. Collected cells were lysed by pipetting the pellet with 750 μL of SDS lysis buffer (50 nM Tris-HCl, pH 8.0, 10 mM EDTA, and 1% SDS) with proteinase inhibitors, followed by incubation on ice for 30 min. The lysed cells were then sonicated using a Sonics VC50 instrument under the following conditions: 10 min of 10 s on/10 s off ice, to shear their DNA to an average size of 350 bp. The antibodies of interest (4 μg) or an IgG-negative control was used in overnight incubations. Precleared protein A/G agarose beads (1:1; Millipore, Billerica, MA, USA) were added to the samples, which were then incubated for 2 h at 4 °C. Beads were washed using four individual buffers as follows: low-salt buffer (0.1% SDS, 0.1% Triton X-100, 150-mM NaCl, 2-mM EDTA, and 20-mM Tris-HCl, pH 8.0); high-salt buffer (0.1% SDS, 0.1% Triton X-100, 500-mM NaCl, 2-mM EDTA, and 20-mM Tris-HCl, pH 8.0); IP wash buffer (0.5 M LiCl, 1% NP-40, 1% deoxycholic acid, and 100 mM Tris-HCl, pH 9.0); and TE buffer (10 mM Tris-HCl pH 8.0, and 1 mM EDTA). Samples were eluted from beads and reverse cross-linked using 0.3 M NaCl at 65 °C overnight. Proteinase K treatment was used to release DNA, and phenol/chloroform/isoamyl alcohol (25:24:1) was used to isolate DNA fragments. Data were collected using a real-time PCR assay. The antibodies used in this experiment were anti-AhR (1:1000, sc-8088), anti-Nrf2 (1:1000, sc-722), anti-Arnt (1:1000, NB100-124), anti-MafK (1:2000, ab229766), IgG (1:1000, C15400001015), and anti-Jdp2 (1:500, from Dr. Aronheim). The primers used to detect each fragment are listed in Supplementary Table S2.

### Construction of AhR promoter plasmids and site-directed mutagenesis

The AhR promoter region was cloned from chromosome 12 of a C57BL/6J *Mus musculus* strain (GRCm38.p4 C57BL/6J 35535598 to 35536615) using a KAPA HiFi PCR kit (Kapa Biosystems, Roche Sequencing Solutions, Pleasanton, CA, USA) with the following primers: 5′–ATAGGTACCGGATCCCCTCTTCTCCTTCT–3′, 5′–ATACTCGAGGCTGCTCATGGTG–3′, with *Kpn*I or *Xho*I added at the 5′ end. Fragments with a total length of 1947 bp were cloned into the pGL4.1 plasmid (Promega). The construct was confirmed using endonuclease restriction analysis and new generation sequencing, to confirm the location and orientation of fragments. The binding sites on the AhR promoter region were predicted using ALGGEN-PROMO (http://alggen.lsi.upc.edu). QuikChange Lightning Site-Directed Mutagenesis Kits (Agilent Technologies, Santa Clara, CA, USA) were used to change individual sites of the AhR promoter regions, as listed in Supplementary Table S3.

### Short hairpin RNA-mediated and small interfering RNA-mediated gene knockdown

Recombinant short hairpin RNA (shRNA) lentiviruses against mouse AhR, Nrf2, MafK, Arnt, GFP, and Jdp2 were obtained from the small interfering RNA (siRNA) Core Center of Academia Sinica (Taipei, Taiwan). Predesigned ON-TARGETplus SMARTpool siRNAs against mouse AhR, ARNT, Ahrr, MafK, and Nrf2 and a control scrambled siRNA were obtained from GE Dharmacon (Austin, TX, USA). MEFs seeded in a 6-well (for western blotting) or 24-well (for luciferase reporter assay) plates were transfected by 20–40 nM of either siRNA or negative-control RNA in a final volume of 0.2 mL (6-well plates) or 0.5 mL (24-well plates) in OPTI-MEM (Invitrogen) with Lipofectamine RNAiMAX (Invitrogen). In the case of shRNAs, MEFs were infected at a multiplicity of infection of 10. After 24 h, the cells were washed in fresh medium containing 10% FBS, transfected with luciferase plasmids, and analyzed using a luciferase reporter assay, as described above. To confirm the knockdown efficiency of shRNAs and siRNAs, cells were harvested at 48 and 72 h after shRNA infection and siRNA transfection, respectively, and analyzed by immunoblotting and other assays. The siRNAs used in this study are as shown in Supplementary Table S4.

### ROS detection by chloromethyl-2′,7′-dichlorofluorescein diacetate fluorescence and flow cytometry

MEFs were cultured in 0.1% gelatin-coated 3-cm Petri dishes. After treatment with 0.1% DMSO for the indicated time, cells were washed with warm Hanks’ balanced salt solution (HBSS: Gibco-Thermo Fisher Scientific) and then loaded with 25-μM chloromethyl-2′,7′-dichlorofluorescein diacetate (CM-H_2_DCFDA; C-6827, Life Technologies) in complete growth medium for 10 min at 37°C in the dark. After loading, cells were washed twice with HBSS and examined using a Nikon inverted fluorescence microscope. Three to five fields were selected for imaging with a 10× objective lens and quantified using ImageJ (National Institutes of Health, Bethesda, MD, USA). The value of WT basal (set as 1.0) was used to normalize the results.

### Analysis of cellular ROS accumulation

The ROS-Glo H_2_O_2_ assay (Promega) was used to analyze the level of ROS. After 2 h of treatment with antioxidants and H_2_O_2_ and washing twice with HBSS, cells were incubated with the ROS-Glo detection solution for 20 min. Fluorescence was detected using a GloMax fluorometer (Promega). In some cases, we measured the net intracellular accumulation of ROS, using the oxidation product of dihydrorhodamine 123 (DHR 123) (Molecular Probes, Eugene, OR, USA). After 2 h of treatment with DMSO, cells were washed twice with HBSS solution and loaded with 10 mmol/l of H_2_DCFDA or DHR 123 in a 5% CO_2_ incubator kept at 37°C. After 5-min incubation and washing twice with HBSS (Gibco), cells were suspended in complete medium and examined under a microscope. The number of DCF-stained cells was measured in an area of 8.75 mm^2^ (Tanigawa et al. 2013).

### MTT assay

Colorimetric MTT [3-(4,5 dimethyldiazol-2-yl)-2,5-diphenyltetrazolium bromide; Merck Promega] assays were carried out to determine the effect of DMSO on the viability of MEFs. Cells were seeded into 96-well plates at a density of 5000 cells/well in 100-μL DMEM medium containing 10% FBS. Cell viability was determined 24 h after treatment with various concentrations of DMSO by adding 10% MTT dye to each well. Plates were incubated at 37°C for 4 h to allow mitochondrial dehydrogenase enzymes in live cells to generate formazan crystals from MTT. After washing, DMSO was added to dissolve the crystals. Finally, after shaking in the dark for 10 min, the absorbance at 550 (A550) and 690 nm (A690) was measured using a microplate reader to determine the percent viability of cells.

### Apoptosis and necrosis assays

The number of apoptotic and necrotic cells was measured by annexin V (AnnV) and propidium iodide (PI) staining and flow cytometry. After treatment, cells were removed using trypsin/EDTA, washed with PBS, and resuspended in AnnV binding buffer (BD Biosciences, Franklin Lakes, NJ, USA). Fluorescein isothiocyanate (FITC)–labeled AnnV and PI (Cat. No. 556547, BD Biosciences) were added to cells and incubated for 15 min. The number of AnnV^+^/PI^−^ and AnnV^+^/PI^+^ events/μL was quantified using an Micro-Plus Flow cytometer (Apogee Flow, Hemel Hempstead, UK). In addition, the apoptotic index was determined by calculating the percentage of AnnV positive cells in at least 500 cells. Cell death was visualized with annexin V-FITC and PI (ab14085; Abcam, Cambridge, UK). Cells were treated with both dyes for 5 min in the dark, fixed, and counterstained with DAPI or 0.8-μg/mL Hoechst 33342. The percentage of apoptotic and necrotic cells was quantified by flow cytometry and then calculated according to the relative ratio of WT and *Jdp2*-KO MEFs.

### Measurement of cell area from the immunofluorescence staining of pMLC2 and assessment of actin stress fibers

MEFs were replated in 8-well chamber slides precoated with 0.1% gelatin and allowed to spread for 2 h at 37°C in complete medium containing DMSO (0.1%). Cells were fixed and permeabilized with 4% formaldehyde for 30 min and 0.2% Triton X-100/PBS for 15 min at room temperature. After blocking with 5% goat serum/PBS, cells were stained overnight with an anti-pMLC2 antibody (1:50, Cell Signaling Technology, Danvers, MA, USA) at 4°C, washed thoroughly with PBS, and incubated with an Alexa Fluor-594 goat anti-pMLC2 secondary antibody (1:400; Invitrogen) at room temperature for 60 min. To label actin stress fibers, actin was counterstained using Alexa Fluor 488–labeled phalloidin (1:80; Invitrogen), which produced green fluorescence. Finally, cell nuclei were stained with DAPI, which produces blue fluorescence. Fluorescence images from five different fields per treatment were acquired using a Nikon epifluorescence microscope (Nikon Instruments, Melville, NY, USA). Cell area and fluorescence intensity were quantified using ImageJ software. The relative intensity was determined by the DAPI setting intensity at 1.0, which was then divided by the value of either phalloidin or pMLC2 fluorescence to obtain the relative ratio. In another set of experiments, MEFs were washed with PBS, fixed with 4% formaldehyde, and processed for F-actin and nuclear staining, as described above. For each treatment, at least five arbitrarily selected fields were acquired.

### Statistical analyses

Results were all presented as the mean ± SEM. Statistical comparisons between experimental conditions were carried out using GraphPad Prism 5.0 software (GraphPad Software, San Diego, CA, USA). For multiple comparisons, a one-way ANOVA followed by a Tukey post hoc test or a two-way ANOVA with a Bonferroni post hoc test was used. An unpaired, two-tailed Student *t*-test was used to compare the control and treatment groups. A paired, one-tailed Student *t*-test was used to determine each site-directed mutagenesis location on the *AhR* promoter. A Mann-Whitney nonparametric median statistical test was used for analyses of cell areas. All differences were estimated statistically significant at *P* < 0.05.

## Results

### Effect of DMSO on cell spreading and F-actin dynamics

AhR is reported to regulate the cell shape, adhesion, and migration of normal MEFs (Mitra et al. 2005; Li et al. 2005; Tsai et al. 2017; Ikuta and Okabe-Kado 2018; Novikov et al. 2016; Qin and Powell-Coffman 2004; Barouki et al. 2007; Mulero-Navarro et al. 2005; Carvajal-Gonzalez et al. 2009).

**Fig. 1:**
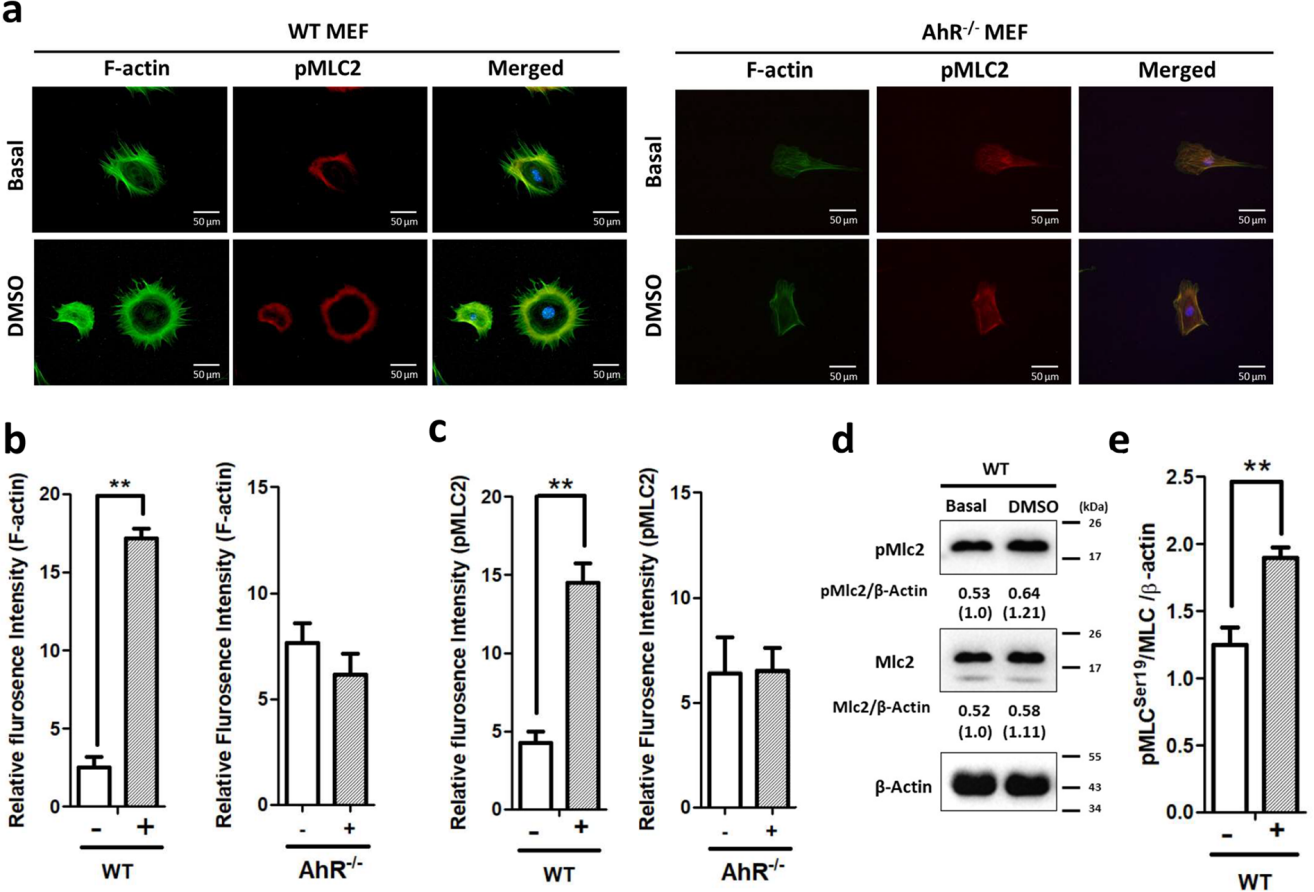
Extension of actin stress fibers and cell spreading of WT and *AhR*^−/−^ MEFs after exposure to DMSO. **a** Cells were starved of serum overnight before DMSO treatment for 24 h and exposed to 0.1% DMSO for 2 h. At the end of treatment, cells were rinsed with PBS, fixed with 4% formaldehyde, and then processed for F-actin staining and phosphorylated myosin light chain staining as described in the “Materials and methods” section. Nuclei were stained with DAPI, as indicated by blue fluorescence and the images with each of the three fluorescent colors merged. **b** Quantification of signaling of F-actin fibers. Five fields were examined, and the fluorescence intensity was quantified. WT MEFs and *AhR*^−/−^ MEFs in the presence and absence of 0.1% DMSO for 2-h treatment. Cell areas with actin fibers were calculated as described in the “Materials and methods” section. Data represent the mean ± SEM (*n* = 5), **P < 0.01. **c** Quantification of signaling of pMLC2. Five fields were examined, and the fluorescence intensity was quantified. WT MEFs and *AhR*^−/−^ MEFs in the presence and absence of 0.1% DMSO for 2-h treatment. Cell areas with pMLC2 were calculated as described in the “Materials and methods” section. Data represent the mean ± SEM (*n* = 5), ** *P* < 0.01. Representative immunoblots **d** and quantitative results **e** for cells harvested 2 h after 0.1% DMSO treatment. Data are presented as mean ± SEM (*n* = 5). All statistical analysis was performed by Student’s *t*-test (** *P* < 0.01)

Initially, we examined the effect of 0.1% DMSO, the control solvent dose, on cell spreading. The fluorescence intensity of F-actin fibers in WT cells in response to 0.1% DMSO was enhanced >6-fold compared with levels in the absence of DMSO (Fig. 1a, b). AhR-depleted MEFs did not exhibit cell spreading of F-actin (Fig. 1b), in line with previous reports (Carvajal-Gonzalez et al. 2009; Taulet et al. 2012). Phosphorylated MLC2 at position serine 19 (pMLC2) is known as a critical component of the actin stress fiber remodeling process (Bisaria et al. 2020). Notably, the pMLC2 fluorescence/signal intensity was >3-fold higher in the presence than in the absence of DMSO in WT MEFs, but depletion of AhR prevented this effect (Fig. 1c). To further examine the effects of 0.1% DMSO on actin cytoskeleton remodeling, western blot analysis was used to determine the relative expression levels of pMLC and MLC per *β*-actin and calculate the ratio of pMLC2 expression in WT MEFs treated with 0.1% DMSO for 2 h. This analysis revealed that the expression of pMLC2/MLC/*β*-actin under steady-state conditions was slightly higher (1.4-fold) in the presence of DMSO (Fig. 1d, e).

Taken together, these results indicate that 0.1% DMSO induced cell spreading of MEFs. To confirm the effect of DMSO on cell spreading, we examined the effect of different concentrations of DMSO on the cytotoxicity and proliferation of WT MEFs.

### Effect of DMSO on cytotoxicity and cell proliferation

Cytotoxicity, necrosis, and apoptosis in MEFs were examined after exposure to DMSO at concentrations ranging from 0.01 to 10.0% (Fig. S1a). An MTT assay demonstrated that the lowest level of cytotoxicity was induced by 0.01–0.1% DMSO (i.e., >85% MTT activity was maintained compared with control), whereas significant cytotoxicity was detected at concentrations ranging from 1.0 to 10.0% DMSO (i.e., <60% of MTT activity was detected). At 10% DMSO, NAD(P)H–dependent cellular oxidoreductase activity reflected by the MTT assay was reduced by 50%. At 0.1% DMSO, which is the concentration routinely used as a control solvent in toxicology studies, >85% of the metabolic processes were retained (Fig. S1a). At concentrations of DMSO >0.1%, significant apoptosis and necrosis were detected (Fig. S1b, c). The apoptosis induced by 0.1% DMSO might be due to the Fas-Fas-L-caspase 8–dependent cascade in light of the higher expression of FAS, FAS-L, Bax, and Caspase 8 (Fig. S2) (Shang et al. 2017). These data indicate that 0.1% DMSO, a control solvent dose, reduced metabolic activity and cell proliferation and induced cytotoxicity. Therefore, we examined the effects of exposure to this concentration of DMSO in further experiments.

### Effect of DMSO on ROS level and AhR protein expression

The significant apoptosis, necrosis, and metabolic activity induced by DMSO suggest that cellular oxidative stress is enhanced by exposure to DMSO in MEFs; therefore, we examined the level of ROS in MEFs exposed to DMSO and observed that it was dose-dependently increased compared with the experimental control.

**Fig. 2:**
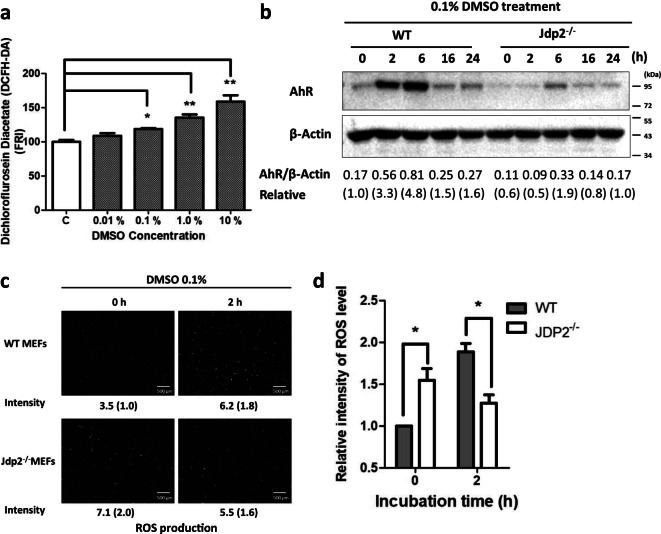
Measurement of ROS activity in WT MEFs in response to DMSO. **a** MEFs incubated with 0 or 0.01, 0.1, 1.0, and 10.0% DMSO for 2 h were stained with 25 micro M CM-H2-DCFDA and examined by flow cytometry as described in the “Materials and methods” section. Data are presented as mean ± SEM (*n* = 5, in triplicate). ROS levels in the control incubation without DMSO were arbitrarily set to 100. (* *P* < 0.05, ** *P* < 0.01 ). **b** Comparison of the levels of AhR protein in WT and *Jdp2*^−/−^ MEFs after incubation with 0.1% DMSO for 0, 2, 6, 16, and 24 h. **c** Comparative ROS activity was measured in WT and *Jdp2*^−/−^ MEFs in response to 0.1% DMSO for 2 h. ROS production was detected using CM-H2DCFDA as described in the “Materials and methods” section. Representative fluorescence images of ROS generation in WT (top) and *Jdp2*^−/−^ (bottom) MEFs are shown. **d** Collective fluorescence images of ROS levels detected using CM-H2DCFDA after treatment with DMSO were analyzed using ImageJ software. The fluorescence of WT MEFs without DMSO exposure is taken as 1.0. Values represent mean ± SEM (*n* = 6). Statistical analysis was performed by two-way ANOVA with Bonferroni posttests (*P < 0.05)

Exposure to 0.1–10% DMSO yielded further significant increases in the level of ROS (Fig. 2a). In WT MEFs, exposure to 0.1% DMSO for 2 and 6 h increased the level of AhR protein expression by 3.3–4.8-fold compared with that observed in the absence of DMSO, followed by a significant reduction in expression at 16 and 24 h (Fig. 2b). However, in *Jdp2*^−/−^ MEFs, no significant increase in AhR protein levels was detected, except after 6-h exposure (1.9-fold) to 0.1% DMSO (Fig. 2b). Thus, we concluded that the induction of AhR expression in WT MEFs was rapid and occurred as early as 2-h posttreatment.

We also examined whether 0.1% DMSO treatment could induce the expression of AhR proteins in other cells. Enhanced expression of AhR protein was also observed in NIH3T3 mouse fibroblast cells, 1.8-fold after 6-h exposure, and in WI38 human diploid cells and HepG2 human hepatoblastoma cells, 2.1-fold and 1.5-fold after 2-h exposure, respectively (Fig. S3a-c). Moreover, the expression of AhR target gene products such as Cyp1a1 and Cyp1b1 was also examined in WT and *Jdp2*-KO MEFs in response to 0.1% DMSO (Fig. S3d). The expression of Cyp1a1 was enhanced about 2-fold by DMSO exposure in WT MEFs (Fig. S3d), but not in *Jdp2*-KO MEFs (data not shown). However, expression of Cyp1b1 was not significantly enhanced by 0.1% DMSO in WT MEFs or *Jdp2*-KO MEFs. In NIH3T3 cells, the expression of Cyp1b1 was significantly enhanced >5-fold after 6-h exposure to 0.1% DMSO, but the expression of Cyp1a1 was not increased (Fig. S3a). In HepG2 cells, the expression of both Cyp1a1 and Cyp1b1 was enhanced 1.8-fold and 2.3-fold after 2-h exposure, respectively. In WI38 cells only, Cyp1a1 was enhanced 1.9-fold after 2-h exposure, but Cyp1b1 expression was not increased (1.2-fold; Fig. S3b and c). Thus, the upregulation of Cyp1a1 and Cyp1b1 might be cell type–specific and dependent on exposure time.

In addition, another phase I ligand, TCDD (10 nM), induced 1.4-fold higher expression of AhR protein than control after 6-h exposure (Fig. S4a). Exposure to 0.1% DMSO for 6 h also enhanced AhR expression by 1.8-fold above control (Fig. S4a), and AhR promoter-luciferase activity was 3-fold and 2-fold higher than the control levels, respectively (Fig. S4b). Thus, the effect of TCDD was more marked than DMSO, but DMSO enhanced AhR expression significantly compared with control levels.

The expressions of AhR target genes are primary regulated by the increases of intracellular ROS levels and endogenous AhR caused by the phase I ligands as reported previously (Katsuoka et al. 2005; Moldogazieva et al. 2018; Wolfle et al. 2014; Fuyuno et al. 2018). Higher ROS levels (more than 1.5-fold) were detected in WT MEFs at 2 h after exposure to 0.1% DMSO, whereas the ROS levels present in *Jdp2*^−/−^ MEFs were significantly reduced by 70% DMSO after 2-h exposure of DMSO, relative to WT MEFs levels (Fig. 2c, d). These results indicate that a Jdp2 deficiency might reverse the effect of a 2-h exposure to 0.1% DMSO on ROS levels.

### Effects of Jdp2, AhR, and Nrf2 on *AhR* promoter activation and apoptosis of MEFs

**Fig. 3:**
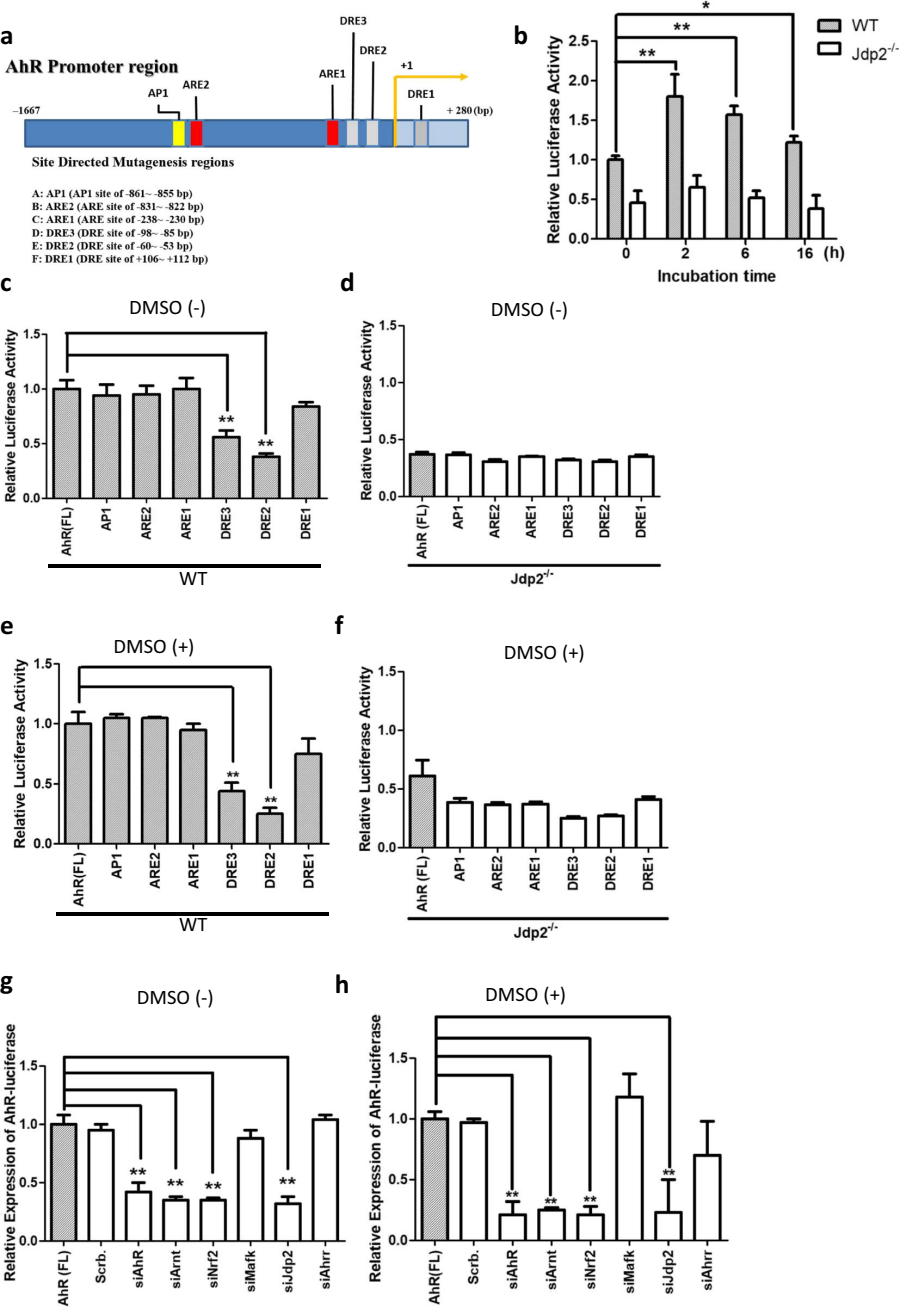
Characterization of *AhR* promoter activity. **a** Schematic representation of the positions of each DRE and ARE in the *AhR* promoter region. Each element is shown at the position from the putative transcription start site, which was mutated to generate the mutants of DRE and ARE. **b** Relative activity of pGL4.1-*AhR*-luciferase in WT and *Jdp2*^−/−^ MEFs treated with 0.1% DMSO for 0, 2, 6, and 16 h. Luciferase activities were calculated as the ratio of the *AhR*-luciferase activity to that of the control pGL4.1 and expressed as relative luciferase activity. Values represent the mean ± SEM of five independent measurements. Statistical analysis was done by two-way ANOVA with Bonferroni posttests (* *P* < 0.05, ** *P* < 0.01). **c–f** Effects of the mutation of each *cis* element, i.e., ARE1, ARE2, DRE1, DRE2, and DRE3, on the *AhR* promoter region. Luciferase activity was measured in WT MEFs (**c**, **e**) and *Jdp2*^−/−^ MEFs (**d**, **f**) in the absence (**c**, **d**) or presence of 0.1% DMSO (**e**, **f**) as described in the “Materials and methods” section. The luciferase activity of full length (FL) *AhR*-luciferase was arbitrarily set to 1.0. Values represent the mean ± SEM (*n* = 5). Statistical analysis was performed by one-way ANOVA with Tukey’s test (** *P* < 0.01). **g**, **h** Effect of siRNAs against each representative transcription factor (AhR, Arnt, Nrf2, MafK, Jdp2, and Ahrr) on pGL4.1-*AhR*-luciferase activity in WT MEFs in the absence (**g**) or presence (**h**) of 0.1% DMSO. The luciferase activity of FL *AhR*-luciferase was arbitrarily set to 1.0. Values represent the mean ± SEM (*n* = 3). Statistical analysis was performed by one-way ANOVA with Tukey’s test (** *P* < 0.01)

Next, we examined whether exposure to DMSO increased the promoter activity of AhR. The promoter of AhR contains three putative DREs and two putative AREs (Fig. 3a). The *AhR* promoter-luciferase reporter (*pAhR-Luc*) was activated in WT MEFs, but not in *Jdp2*^−/−^ MEFs after 2 h exposure to 0.1% DMSO (Fig. 3b), and thereafter, *AhR* promoter activity was gradually reduced during the following 14 h.

**Fig. 4:**
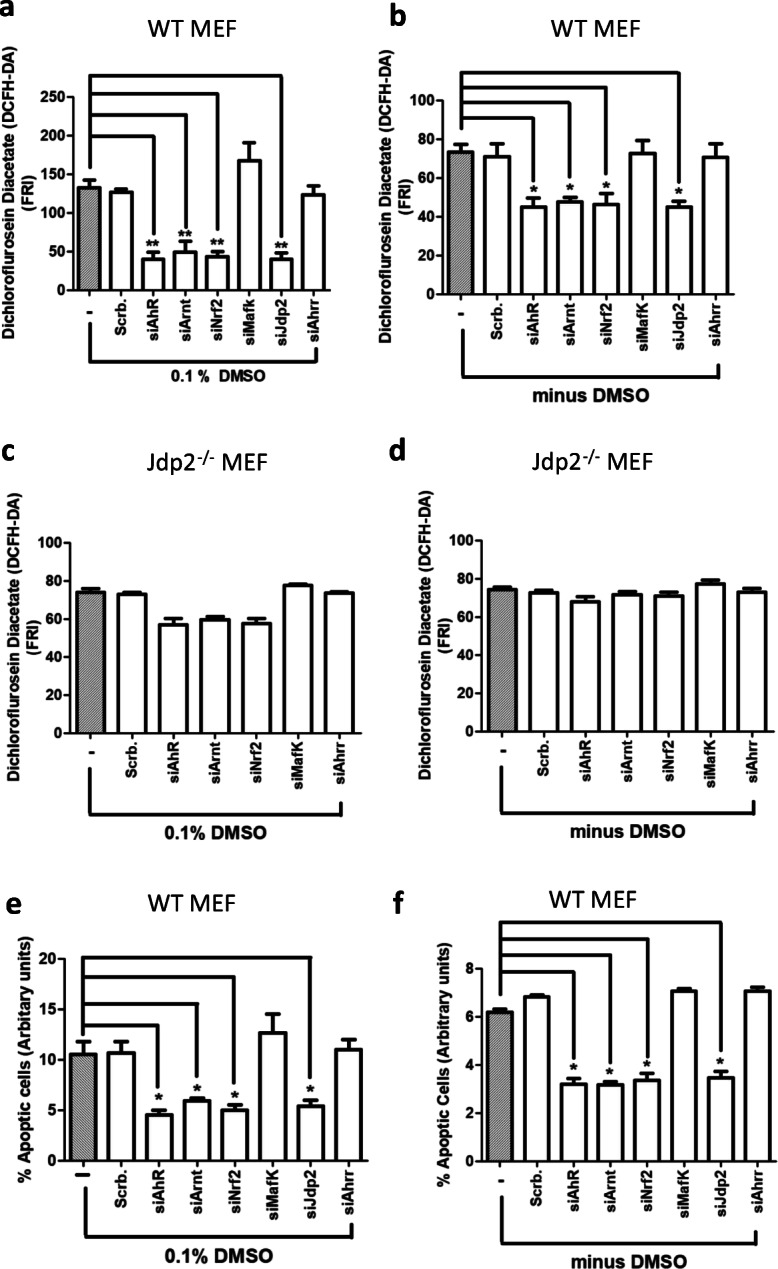
Regulation of production of ROS and apoptotic activity in WT and *Jdp2*^−/−^ MEFs by siRNA against AhR, Arnt, Ahrr, Nrf2, Jdp2, and MafK. **a–d** WT (**a**, **b**) and *Jdp2*^−/−^ MEFs (**c**, **d**) incubated with various siRNAs against AhR, Arnt, Nrf2, Ahrr, Jdp2, and MafK without (**b**, **d**) and with 0.1% DMSO (**a**, **c**) for 2 h were stained with 0.25 M CM-H2DCFDA and examined by flow cytometry as described in the “Materials and methods” section. Data are presented as mean ± SEM (*n* = 3, in triplicate). Statistical analysis was done by one-way ANOVA with Tukey’s test (* *P* < 0.05, ** *P* < 0.01 ). **e**, **f** Effect of siRNA against each representative transcription factor (AhR, Arnt, Nrf2, MafK, Jdp2, and Ahrr) on the apoptotic activity in WT MEFs in the presence (**e**) and absence (**f**) of 0.1% DMSO. Values represent the mean ± SEM (*n* = 3). Statistical analysis was performed by one-way ANOVA with Tukey’s test (*P < 0.05)

Both DRE2 and DRE3 were critical for the activation of p*AhR*-Luc because these mutants of DRE2 and DRE3 *cis* elements decreased *AhR* promoter activity to 65 and 38% in WT MEFs in the absence of 0.1% DMSO, respectively (Fig. 3c); and in the presence of 0.1% DMSO, both DRE2 and DRE3 mutations also reduced the *AhR* promoter activity to 45 and 26%, respectively (Fig. 3e). In *Jdp2*^−/−^ MEFs, we did not detect any effects of DRE2 and DRE3 mutations in the presence or absence of 0.1% DMSO (Fig. 3d, f). Regardless of the presence or absence of 0.1% DMSO, knockdown of AhR, Arnt, Nrf2, and Jdp2 decreased *AhR* promoter activity. By contrast, knockdown of MafK and Ahrr and a scrambled siRNA did not suppress the *AhR* promoter activity in WT MEFs (Fig. 3g, h, Fig. S5). Knockdown of AhR, Arnt, Nrf2, and Jdp2, but not MafK and Ahrr, also decreased ROS levels (Fig. 4a, b) and apoptosis (Fig. 4e, f) in WT MEFs in the presence or absence of 0.1% DMSO. In studies of *Jdp2*^−/−^ MEFs, we did not observe a significant effect of the various siRNAs on *AhR* promoter activity, ROS levels (Fig. 4c, d), or apoptosis activity (Pan et al. 2010). These results suggest that Jdp2 and Nrf2, as well as AhR-Arnt, contribute to the activation of the *AhR* promoter and regulate ROS levels and cell apoptosis.

### Recruitment of Jdp2 and *trans* factors to the DRE and ARE sites of the *AhR* promoter

**Fig. 5:**
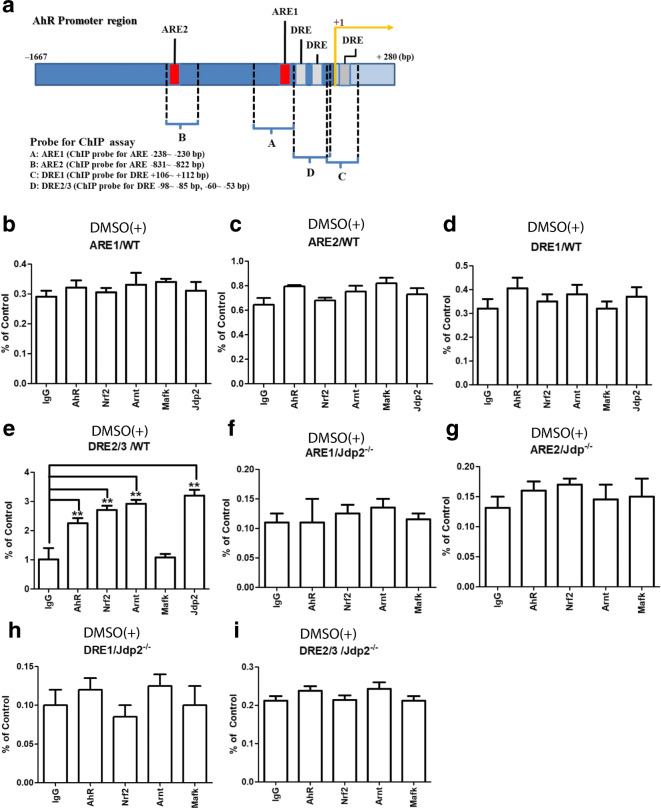
Differential recruitments of transcription factors such as AhR, Arnt, Nrf2, and MafK to ARE and DRE sites in WT and *Jdp2*^−/−^ MEFs. **a** Schematic representation of the mouse *AhR* promoter and the position of *cis* elements, such as ARE1, ARE2, DRE1, and DRE2/3, which were detected in the ChIP assay. **a–d**; **f–h** The regions that were amplified by PCR with the specific corresponding primers (ARE1, ARE2 and DRE1) and (**e**, **i**) with the primers that contained the DRE 2 and 3 *cis* elements were indicated in WT MEFs. The ChIP-qPCR analyses were performed using chromatin extracts from WT (**b–e**) and *Jdp2*^−/−^ MEFs (**f–i**) with the indicated antibodies and normal IgG (as a negative control). The probes of ARE1 (**b**, **f**), ARE2 (**c**, **g**), DRE1 (**d**, **h**), and DRE2/3 (**e**, **i**) are shown in the presence of 0.1% DMSO, respectively. The values in the absence of DMSO are detailed in Fig. S6. Values represent mean ± SEM (*n* = 5). Statistical analysis was performed by one-way ANOVA with Tukey’s test (** *P* < 0.01)

Next, we performed chromatin immunoprecipitation (ChIP)-qPCR assays to confirm the interaction between the identified *trans* factors (AhR, Arnt, Nrf2, MafK, and Jdp2) and the *cis* elements (ARE and DRE) of the *AhR* promoter. Four pairs of qPCR primers were used to detect the recruitment of these *trans* factors to the AhR promoter (Fig. 5a). After 2 h of exposure of WT MEFs to 0.1% DMSO, the ARE1 and ARE2 sites and the DRE1 site were not functional (Fig. 5b–d). On the contrary, Jdp2, Nrf2, AhR, and Arnt were recruited to the DRE2/3 sites (Fig. 5e), which are critical for DMSO-induced *AhR* promoter activity. In *Jdp2*^−/−^ MEFs, none of these transcription factors were recruited to the ARE and DRE sites of the AhR promoter (Fig. 5f–i). In the absence of 0.1% DMSO, we did not detect specific recruitment of any transcription factors in WT or *Jdp2*^−/−^ MEFs (Fig. S6a–h).

**Fig. 6:**
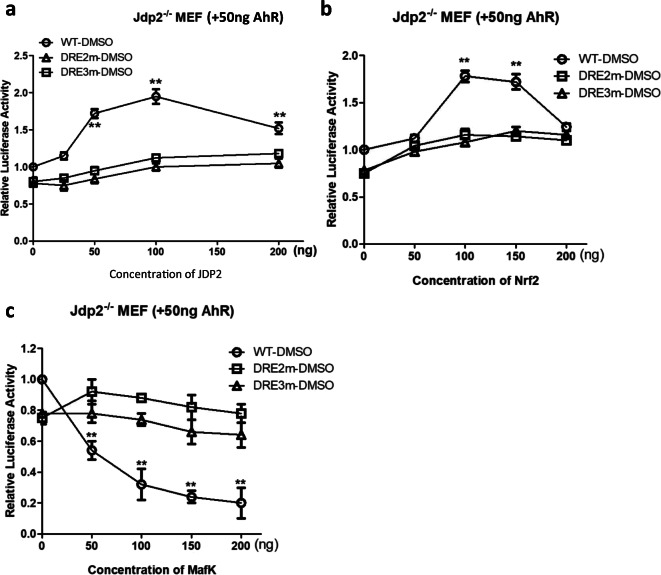
Effect of Jdp2, MafK, and Nrf2 on AhR-luciferase activity via DRE2 and DRE3 in *Jdp2*^−/−^ MEFs. **a** AhR-luciferase, DRE2 mutant-luciferase, and DRE3 mutant-luciferase (50 ng, respectively) plus 0–200 ng of *pcDNA-Jdp2* were transfected into *Jdp2*^−/−^ MEFs. One day after transfection, 0.1% DMSO was added for 2 h, cells were collected, and luciferase activity was measured as described in the “Materials and methods” section. **b** *AhR*-luciferase, DRE2 mutant-luciferase, and DRE3 mutant-luciferase (50 ng, respectively) plus 0–200 ng of *pcDNA-Nrf2* were transfected into *Jdp2*^−/−^ MEFs. One day after transfection, 0.1% DMSO was added for 2 h, cells were harvested, and luciferase activity was measured as described in the “Materials and methods” section. Data represent the mean ± SEM (*n* = 5). **c** AhR WT-luciferase, DRE2 mutant-luciferase, and DRE3 mutant-luciferase (50 ng, respectively) plus 0–200 ng of *pcDNA-MafK* were transfected into *Jdp2*^−/−^ MEFs. One day after transfection, 0.1% DMSO was added for 2 h, cells were collected, and luciferase activity was measured as described in the “Materials and methods” section. All statistical analysis was performed by two-way ANOVA with Bonferroni posttests (* *P* < 0.05, ** *P* < 0.01)

Based on these results, we focused on the effects of Jdp2, Nrf2, and MafK on regulating the AhR promoter through DRE2 and DRE3. In *Jdp2*^−/−^ MEFs, the forced expression of Jdp2 (Fig. 6a) or Nrf2 (Fig. 6b) increased *AhR* promoter activity in the presence of DMSO. However, the AhR promoter mutants of the DRE2 and DRE3 sites were not activated by the overexpression of Jdp2 (Fig. 6a) or Nrf2 (Fig. 6b). By contrast, the ectopic expression of MafK decreased AhR promoter activity, and, again, both the DRE2 and DRE3 mutants were without effect (Fig. 6c).

### Association of AhR and Nrf2 with Jdp2 in vivo

**Fig. 7:**
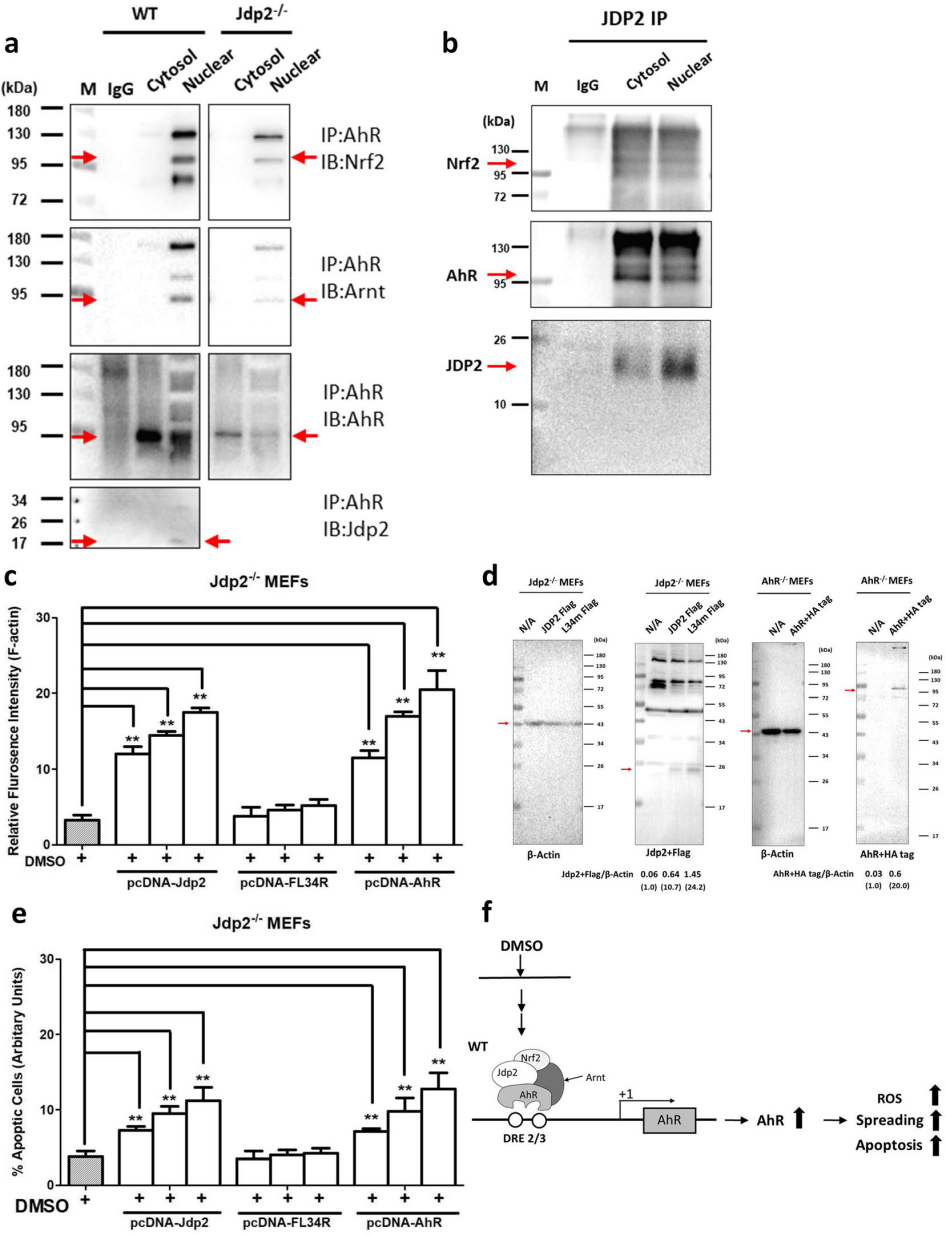
Jdp2 interacts with AhR and Nrf2 in nuclei and rescue of *JdP2*^*−/−*^ MEFs by AhR. **a** Cell lysates (300 μg) from cytosolic and nuclear fractions of WT and *Jdp2*^−/−^ MEFs were immunoprecipitated with antibodies against AhR, and the bound proteins were blotted with antibodies against Nrf2, Arnt, AhR, and Jdp2, as described in the “Materials and methods” section. IgG, as a negative control. **b** Cell lysates (300 μg) from cytosolic and nuclear fractions from WT MEFs were immunoprecipitated with anti-Jdp2 antibodies, and the bound proteins were blotted with anti-Nrf2, anti-AhR, and anti-Jdp2 antibodies, as described in the “Materials and methods” section. IgG was used as a negative control. **c** Rescue of DMSO-induced cell spreading by overexpression of Jdp2, its mutant, and AhR. Rescue of 2-h DMSO-induced cell spreading (**c**) and apoptosis (**e**) by increasing doses (50, 100, and 200 ng) of *pcDNA-Flag-Jdp2*, *pcDNA-FlagJdp2-FL34R*, and *pcDNA-HA-AhR* in *Jdp2*^−/−^ MEFs. The fluorescence intensity in *Jdp2*^−/−^ MEFs without DMSO exposure was set as 1.0. Data represent the mean ± SEM (*n* = 5; ** *P* < 0.01). **d** Representative results of western blot for the tagged proteins of Jdp2, Jdp2FL34R mutant, and AhR in *Jdp2*^−/−^ MEFs transformants by *pcDNA-Flag-Jdp2*, *pcDNA-FlagJdp2-FL34R*, and *pcDNA-HA-AhR*, respectively. **f** Schematic representation of DMSO-induced *AhR* activation through the complex of AhR, Nrf2, and Jdp2 to increase ROS production, cell spreading, and apoptosis in WT MEFs. In *Jdp2*^−/−^ MEFs, only a residual amount of AhR-Arnt was recruited to the DRE2 and DRE3 elements of the *AhR* promoter.

In studies to explore the potential interaction between these *trans* factors, the endogenous association of AhR with Jdp2 or Nrf2 was examined by coimmunoprecipitation and western blotting assays. The results revealed that Nrf2 was coimmunoprecipitated by the anti-AhR antibody in the nucleus of WT and *Jdp2*^−/−^ MEFs (Fig. 7a). The AhR-Nrf2 and AhR-Arnt complexes were detected at levels that were 5- to 7-fold lower in *Jdp2*^−/−^ MEFs than in WT MEFs. The AhR and Nrf2 proteins were coprecipitated by the anti-Jdp2 antibody (Fig. 7b). The colocalization of AhR-Jdp2 was examined by immunofluorescence staining (Fig. S7a). Colocalization of AhR and Jdp2 was detected, even in the absence of DMSO. In addition, the colocalization of Ahr and Arnt was detected as a control (Fig. S7b). In the presence of 0.1% DMSO, this colocalization was also observed (unpublished data). Moreover, the nuclear localization of AhR protein was enhanced by 2-h exposure to 0.1% DMSO or TCDD (unpublished data).

### Jdp2 deficiency decreased cell spreading and AhR can rescue Jdp2-mediated apoptosis

The effects of altered AhR expression and Jdp2 deficiency on cell spreading and apoptosis in response to 0.1% DMSO were examined. The cell spreading activity as measured by F-actin fluorescence intensity, and pMLC2 expression was reduced by 2.5- to 3-fold and 4- to 5.6-fold in *Jdp2*^−/−^ MEFs compared with that of WT MEFs, respectively, with or without 0.1% DMSO (Fig. S8a–d).

To rescue this decreased activity in *Jdp2*^−/−^ MEFs, overexpression of AhR and Jdp2 was performed. Overexpression of AhR or Jdp2 resulted in enhanced cell spreading activity in *Jdp2*^−/−^ MEFs (Fig. 7c, d). However, cell spreading activity was not enhanced by the Jdp2 mutant, Jdp2FL34R, which did not bind to the AP1/ATF *cis* element (Jin et al. 2006) and DRE in vitro (Fig. S9). Similarly, apoptosis was induced by DMSO and the overexpression of Jdp2 and AhR increased apoptosis in a dose-dependent manner; however, the Jdp2FL34R did not reduce the DMSO-induced apoptotic activity (Fig. 7e). In mouse NIH3T3 cells, the similar level of apoptosis induced by 0.1% DMSO was also significant (Fig. S3e and f).

These data suggest that the AhR protein is a downstream target of Jdp2, and the Jdp2-AhR axis is involved in DMSO-induced cell spreading and apoptosis.

## Discussion

The present study produced several key insights into the effects of DMSO exposure on MEFs. First, DMSO at a concentration of 0.1% (v/v; the concentration routinely used as a solvent of organic amphiphilic reagents) induced the key transcription factor, AhR, for phase I enzyme family induction and triggered oxidative stress to generate ROS, which played critical roles for cell migration and apoptosis. Second, in MEFs, the *AhR* promoter was markedly activated by DMSO, even at a low 0.1% concentration. Third, *AhR* promoter activation was triggered by Nrf2 and Jdp2, and by AhR-Arnt, which play a critical role as coupling factors for the “AhR target gene batteries” (Tanigawa et al. 2013). Activation of the *AhR* promoter and ROS induction were detected as early as 2 h after DMSO treatment and subsequently triggered cell spreading and apoptosis in MEFs. Therefore, even at low concentrations, DMSO induced oxidative stress and activated the *AhR* promoter in MEFs. This finding is critical because MEFs derived from specific recombinant KO mice are often used to examine the functions of the deleted gene.

In such studies, many drugs are dissolved in 0.1% DMSO, and thus, in some instances, the experimental results of these experiments may stem from the mixed effects of DMSO and the tested reagents. We therefore recommend researchers use a control that avoids the effect of DMSO itself. A higher concentration of DMSO (2%) stopped the proliferation of rat hepatocytes, which gradually differentiated (Mizuguchi et al. 1998), and the expression of both Cyp3a1 and Cyp4a1 was also enhanced in rat primary hepatocytes (Su and Waxman 2004). In both cases, the concentration of DMSO was higher than 0.1%, but the effects of DMSO were evident.

More recently, DMSO exposure was shown to arrest embryonic development at the 2-cell, 4-cell, and morula stages in a concentration-dependent manner (Kang et al. 2017). Endoplasmic reticulum stress induced by DMSO increased Ca^2+^ levels in mitochondria, mitochondrial depolarization/dysfunction, apoptosis, and autophagy. Moreover, DMSO decreased the number of inner cell masses and trophectoderm and inhibited implantation. A recent study cautioned that with DMSO concentrations ≥1%, the induced oxidative stress was critical in 3T3-L1 adipocytes (Dludla et al. 2018). By contrast, 0.01% DMSO improved the GSH level of 3T3-L1 adipocytes and had minimal effects on cell viability, apoptosis, and/or necrosis (Dludla et al. 2018). Thus, the concentration of DMSO is critical for its effects on oxidative stress and the antioxidation reaction.

However, the precise molecular mechanism by which DMSO induces AhR activation is not known. DMSO can compete for surface hydration water to weaken its attraction to lipid headgroups of lipid vesicles or plasma membranes (Cheng et al. 2015). Lower concentration of DMSO used as a solvent also induces gross changes in macromolecules such as proteins, lipids, and nucleic acids (Tuncer et al. 2018). Thus, AhR might be included in this category. Further studies to clarify whether DMSO is a direct ligand of AhR are essential.

The present study demonstrated that the transcription of AhR was regulated by Nrf2 and Jdp2. Gene batteries of both AhR and Nrf2 seem to be critical for *AhR* expression. Jdp2 appears to be a coupling factor for the phase I and II enzyme batteries and for their functions in ROS homeostasis (Fig. 7f). We reported earlier that Jdp2 is a stimulator of antioxidation involving AREs and complexes with Nrf2 and MafK (Tanigawa et al. 2013). In the present study, we demonstrated that Jdp2 is a stimulator of the *AhR* promoter, which is involved in the control of ROS levels and apoptosis in response to DMSO.

It is unclear how Jdp2 interacts with AhR to maintain the balance between oxidative stress and the redox control (Yamamoto et al. 2018). The *AhR* battery includes both phase I (CYP1A1, 1A2, 1B1) and phase II (e.g., NQO1, GSTA2, and UGT1A6) enzymes (Wang et al. 2008). Yeager et al. (2009) demonstrated that Nrf2 plays a role in the TCDD-mediated enhancement of the classical *AhR* battery genes *Nqo1*, *Ugt*, and *Gst* in mouse liver and that both are needed in succession, but are not sufficient by themselves, to induce the drug-detoxification genes in vivo. A ChIP assay performed using mouse liver cells revealed that AhR binds directly to the DRE-like sequence located in the 5′-untranslated region of *Nrf2* (Miao et al. 2005). Thus, AhR and Nrf2 communication occurs frequently in particular cells and in the presence of some ligands. TCDD enhanced the interaction between AhR and Nrf2 and between AhR and Keap1, as well as the AhR-Arnt interaction (Wang et al. 2013). AhR- and Nrf2-mediated gene expression with distinct respective *cis* elements might be linked to the overlapping responses designated as the “AhR-Nrf2 gene battery” (Yeager et al. 2009). Similarly, the presence of DREs and AREs within the *Nrf2* promoter implicates AhR-Arnt recruitment in the regulation of the antioxidant defense system mediated by Nrf2 (Miao et al. 2005), whereas Nrf2 regulates AhR expression (Shin et al. 2007).

However, the interaction between AhR and Nrf2 in DREs within the *AhR* promoter as the first stage of mediating gene expression has not been clarified. Therefore, we focused on the role of mediator genes, such as *Jdp2*, in the ligand-specific activation of the *AhR* promoter in cooperation with the Nrf2 complex. Here, to our knowledge, we have provided the first reported evidence that DMSO, acting as an AhR agonist, induced the activation of Jdp2, which then associated with the AhR and Nrf2 components and recruited these complexes to the DREs of the AhR promoter. Jdp2 appeared to play a critical role in the activation of the AhR gene (Figs. 3–5). In our study, at 2 h after exposure to DMSO, the DRE2 and DRE3 elements located at the *AhR* promoter were critical for determining the specificity of DMSO actions (Fig. 3c, d). These findings suggest that *AhR* promoter activity is determined by the axis formed by the AhR and Nrf2 proteins, which are connected by Jdp2.

In the phase I response, the AhR promoter is activated by the Nrf2-Jdp2 axis. The DNA-binding mutant FL34R (114 and 121) of Jdp2 cannot associate with the AhR protein in vitro. Therefore, Jdp2 is the likely mediator molecule of the AhR and Nrf2 batteries and responsible for the control of the DMSO-induced activation of the AhR promoter. The phase I ligands also produced the similar regulation of AhR and Nrf2 through Jdp2 (unpublished data). Cell migration is the initial step of the cell invasion and metastasis spread that occurs during cancer development (Barbarov et al. 2015). Bone marrow–derived cells (BMDCs) secrete chemokines such as CCL5, which potentiate metastasis; however, *Jdp2*^−/−^ BMDCs do not induce a capacity for invasion in Lewis lung carcinoma cells (Barbarov et al. 2015). In MEFs, in response to DMSO, the action of the Jdp2-mediated activation of the *AhR* promoter involves cell spreading and migration, followed by apoptosis. Therefore, to prevent oxidation and antioxidation, drugs directed against Jdp2 may represent a way to target the control of apoptosis, allergy, and immune regulation, as part of an immunotherapy.

## Supplementary Information

ESM 1 (DOCX 3011 kb)

## Data Availability

All relevant data and materials are available from the authors upon reasonable request.

## Abbreviations

AhR: aryl hydrocarbon receptor
ARE: antioxidative response element
DMSO: dimethyl sulfoxide
DRE: dioxin-responsive element
Jdp2: Jun dimerization protein 2
MEF: mouse embryonic fibroblasts
Nrf2: nuclear factor (erythroid-derived 2)–like 2
ROS: reactive oxygen species
TCDD: 2,3,6,8-tetrachlorodibenzo-p-dioxin
