# Supplementary material for: Dimethyl sulfoxide stimulates the AhR-Jdp2 axis to control ROS accumulation in mouse embryonic fibroblasts
Source: Cell Biol Toxicol. 2021 Mar 15;38(2):203–22. doi: 10.1007/s10565-021-09592-2 (PMC8986748; doi:10.1007/s10565-021-09592-2)
Supplement: Supplementary file 1 — (DOCX 3011 kb) [file 10565_2021_9592_MOESM1_ESM.docx]

**Supplementary Information**

**Dimethyl sulfoxide stimulates the AhR-Jdp2 axis to control ROS accumulation in mouse embryonic fibroblasts**

Kenly Wuputra^1‒3^, Ming-Ho Tsai^1‒3^, Kohsuke Kato^4^, Ya-han Yang^5^, Jia-Bin Pan^1‒3^, Chia-Chen Ku^1‒3^, Michiya Noguchi^6^, Shotaro Kishikawa^7^, Koji Nakade^7^, Hua-Ling Chen^8^, Chung-Jung Liu^11^, Yukio Nakamura^6^, Kung-Kai Kuo^9^, Ying-Chu Lin^10^, Deng-Chyang Wu^2,11^, Ming-Feng Hou^12^, Shau**-**Ku Huang^8*^, Chang-Shen Lin^1,13*^, and Kazunari K. Yokoyama^1-3,5*^

^1^Graduate Institute of Medicine, ^2^Regerative Medicine and Cell Therapy Research Center, ^3^School of Medicine, ^10^School of Dentistry, Kaohsiung Medical University, Kaohsiung, Taiwan. ^5^Cell Therapy and Research Center, ^9^Department of Surgery, ^11^Department of Gastroenterology, ^12^Department of Obstetrics and Gynecology, Kaohsiung Medical University Hospital, Kaohsiung, Taiwan, ^4^Department of Infection Biology, Graduate School of Comprehensive Human Sciences, the University of Tsukuba, Tsukuba, Japan. ^6^Cell Engineering Division, ^7^Gene Engineering Division, RIKEN BioResource Research Center, Tsukuba, Ibaraki, Japan. ^8^Division of Environmental Health and Occupational Medicine, National Health Research Institutes, 115 Zhunan, Taiwan, ^13^Deaprtment of Biological sciences, National Sun Yat-sen University, Kaohsiung, Taiwan.

*Authors to whom correspondence should be addressed; Shau**-**Ku Huang, Chang-Shen Lin and Kazunari K. Yokoyama (Graduate Institute of Medicine, Kaohsiung Medical University),Tel; ;886-07312-1101, ext. 2729, FAX +886-7313-3849, e-mail; [skhuang@nhri.org.tw](mailto:skhuang@nhri.org.tw); [changshen.lin@kmu.edu.tw](mailto:changshen.lin@kmu.edu.tw); [kazu@kmu.edu.tw](mailto:kazu@kmu.edu.tw))

**[Contents]**

1. **Figure legends of Supplementary Figures (.docx)**
2. **Supplementary Figure 1 to 9 (.png)**
3. **Supplementary Table 1 to 4 (.docx)**
4. **Supplementary figure legends**

**Supplementary Fig. 1.** Effect of DMSO concentration on the NAD(P)H-dependent cellular oxidoreductase activity in the 3-(4,5-dimethylthiazol-2-yl)-2,5-diphenyltetrazolium bromide (MTT) assay, apoptosis, and necrosis in WT MEFs. Effect of 0%, 0.01%, 0.1%, 1.0%, and 10% DMSO on relative absorbance (A_550_‒A_690_; NAD(P)H-dependent cellular oxidoreductase activity) (a), apoptosis (b), and necrosis (c) as described in the Materials and methods. The activity of WT MEFs without DMSO exposure was set as 1.0 (a), and 100% (b, c). Data represent the mean ± SEM (n = 5). Statistical analysis was performed by one-way ANOVA with Tukey's test (* *p*< 0.01, ** *p* < 0.01).

**Supplementary Fig. 2** Expression of FAS/FAS-L-dependent cascade proteins in apoptotic pathways in WT and *Jdp2*^−/−^ MEFs with (+) or without (‒) 0.1% DMSO. Western blots of proteins in Fas/Fas-L pathways were performed as described in the Materials and methods section. Expression of FAS/FAS-L in was enhanced, followed by increased Bax and caspase 8 expression in *Jdp2*^−/−^ MEFs and the addition of 0.1% DMSO increased these expression levels in WT further, but not in *Jdp2*^−/−^ MEFs. Relative expression based on the beta-actin expression was shown in the parenthesis.

**Supplementary Fig. 3.** Effects of 0.1% DMSO on expression of AhR, Cyp1a1, Cyp1b1 and β-actin proteins and apoptosis in NIH3T3 cells. Effect of 0.1% DMSO on the expression of AhR, Cyp1a1, Cyp1b1 and β-actin proteins in mouse fibroblast cells‒NIH3T3 (a), human hepatoblastoma cells-HepG2 (b) and human diploid lung fibroblast cells-WI38 (c) in the presence and absence of 0.1% DMSO. In MEFs, the expressions levels of AhR target proteins Cyp1a1 and Cyp1b1 were shown (d). The relative expression levels are calculated based on theβ-actin level as described in the Materials and method section. The relative expression levels were shown in parentheses. (e) The apoptotic assays stained by annexin V staining was performed as described in the Materials & method section in NIH3T3 cells in the presence or absence of 0.1% DMSO. Values represent the mean ± SEM of three independent measurements. Data represent the mean ± SEM (n = 3). Statistical analysis was performed by one-way ANOVA with Tukey's test (*** *P* < 0.005).

**Supplementary Fig. 4**. Effects of TCDD on expression of AhR protein and AhR-luciferase activity in WT and *Jdp2*^-/-^ MEFs. (a) Expression of AhR protein in WT and *Jdp2*^-/-^ MEFs in response to 10 nM TCDD and 0.1% DMSO for 6 h, as described in Materials and methods section. The relative expression levels were calculated in parentheses. (b) AhR-luciferase activities were quantified as described in the Materials and methods section. Relative activity of pGL4.1-*AhR*-luciferase in WT and *Jdp2*^-/-^ MEFs treated with 10 nM TCDD (left panel) and 0.1% DMSO (right panel) for 6 h. Luciferase activities were calculated as the ratio of the *AhR*-luciferase activity and that of control pGL4.1. Values represent the mean ± SEM of five independent measurements. Statistical analysis was performed by one-way ANOVA with Tukey's test (* *P* < 0.05).

**Supplementary Fig. 5.** Effects of siRNAs against AhR, Arnt, Nrf2, MafK, Jdp2, and Ahrr on AhR target protein expression in WT MEFs. Cells were incubated with siRNAs for 48 h. siRNA-nontreated (–) and -treated (+) cell lysates were blotted to detect each protein, as described in the Materials and methods section. Arrows indicate the positions of target proteins.

**Supplementary Fig. 6.** Regulation of AhR promoter activity in WT and *Jdp2*^−/−^ MEFs by AhR, Arnt, Nrf2, Jdp2, and MafK in the absence of DMSO. The ChIP and RT–PCR assays were conducted using chromatin extracts from WT and *Jdp2*^−/−^ MEFs and the indicated antibodies and normal IgG (as a negative control). The probes for ARE1 (a, e), ARE2 (b, f), DRE1 (c, g), and DRE2/3 (d, h) in WT MEFs (a‒d) and *Jdp2*^−/−^ MEFs (e‒h) are shown in the absence of 0.1% DMSO. Values represent the mean ± SEM (n = 5).

**Supplementary Fig. 7.** Characteristics of Jdp2 activity with AhR. (a) Colocalization of JDP2 and AhR. WT C57/BL6 MEF cells were stained with rabbit anti-Jdp2 (Santa Cruz Biotechnology) and anti-mouse anti-AhR (Clone A-3; Santa Cruz Biotechnology) antibodies and with goat anti-mouse IgG Alexa Fluor 488 and goat anti-rabbit IgG Alexa Fluor 594 (Thermo Fisher Scientific). (b) Colocalization of AhR with Arnt. MEFs were stained with mouse anti-AhR antibody (GeneTex) and rabbit anti-mouse Arnt (GeneTex) and with anti-mouse Alexa Fluor 488 and anti-rabbit Alexa Fluor 594. **(c)** Immunostaining with normal mouse IgG with anti-mouse 488. Scale bars, 30 μm.

**Supplementary Fig. 8.** Extension of cell spreading of WT and *Jdp2^−/−^* MEFs after exposure to DMSO. (a) *Jdp2****^−/−^*** MEFs were starved of serum overnight before DMSO treatment for 24 h and exposed to 0.1% DMSO for 2 h. At the end of the treatment, cells were rinsed with PBS, fixed with 4% formaldehyde, and processed for F-actin and phosphorylated myosin light chain (pMLC2) staining, as described in the Materials and methods section. (b)Western blotting of pMLC2, and MLC2 in basal and 0.1% DMSO-treated (2 h) *Jdp2*^−/−^ MEFs. (c) Quantification of F-actin staining. Five fields of F-actin fibers were examined, and the fluorescence intensity was quantified by Image J software. Data represent the mean ± SEM (n = 5), **P* < 0.05. Quantitative results for cells harvested 2 h after 0.1% DMSO treatment. (d) Quantification of signaling of phosphorylated myosin light chain. Five fields of phosphorylated myosin light chain were examined, and the fluorescence intensity was quantified. Data represent the mean ± SEM (n = 5), **P* < 0.05. Quantitative results for cells harvested 2 h after 0.1% DMSO treatment.

**Supplementary Fig. 9.** Electrophoretic mobility-shift assays (EMSAs) with in vitro recombinant GST-Jdp2 mutant and WT proteins. Interaction of Jdp2 and AhR *in vivo* and Jdp2 leucine zipper mutant did not bind the DRE *cis-*element. Recombinant GST fusion proteins from WT GST-Jdp2 and mutant GST-Jdp2LZ3,4, were prepared and incubated with 0.1 pmol of mouse AhR oligodeoxynucleotide probe (DRE2 cis-element) and 400 ng of each recombinant protein for 20 min at room temperature and then EMSAs were performed as described in the Materials and methods section. The shifted protein‒DNA complexes were identified as bands and the free DNA probes were identified. Lane 1, no protein; lanes 2 and 3, 200 ng of Jdp2 LZ34; and lanes 5 and 6, 200 ng of WT Jdp2.

1. **Supplementary Figures**

**
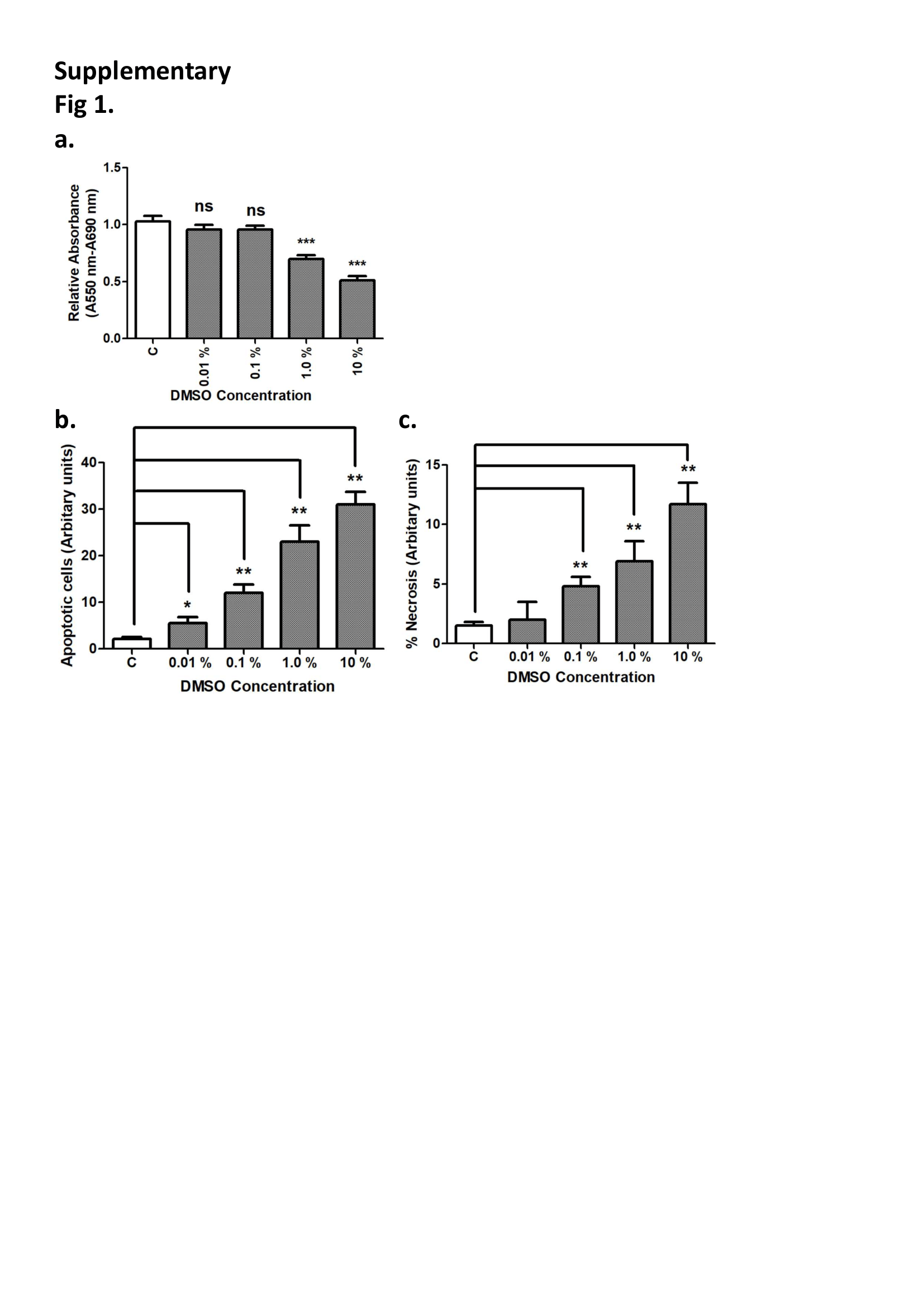
**

**
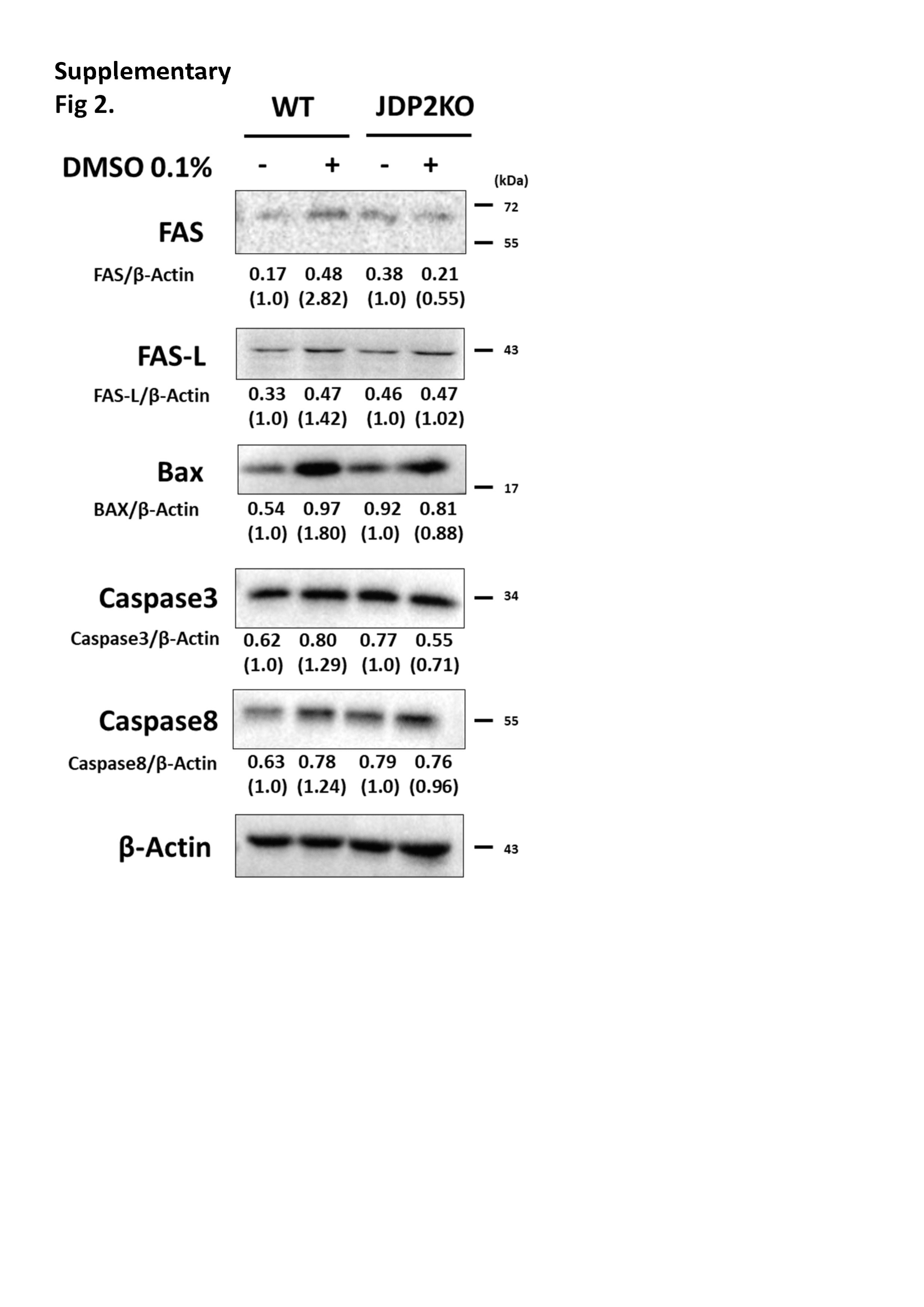
**

**
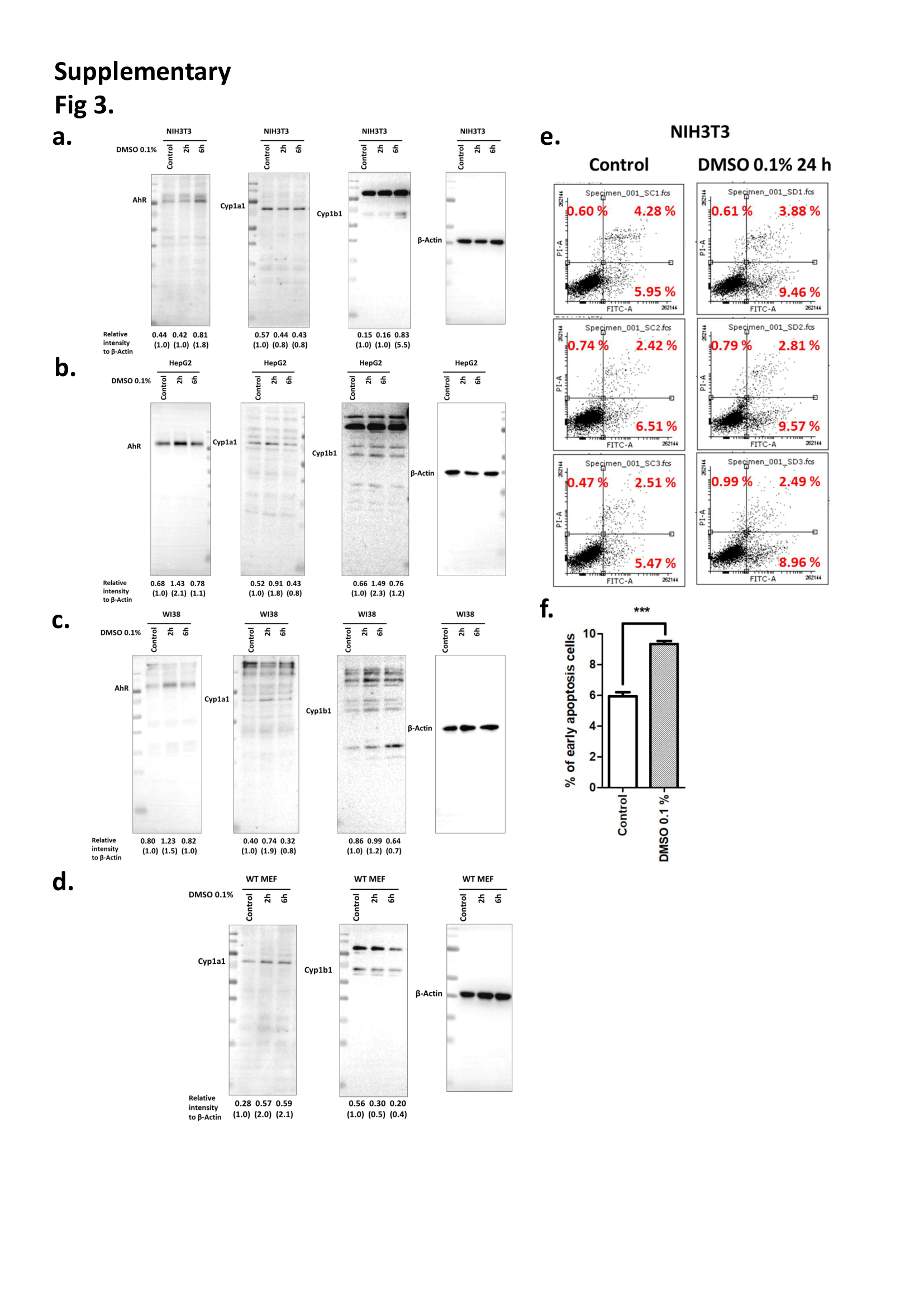
**

**
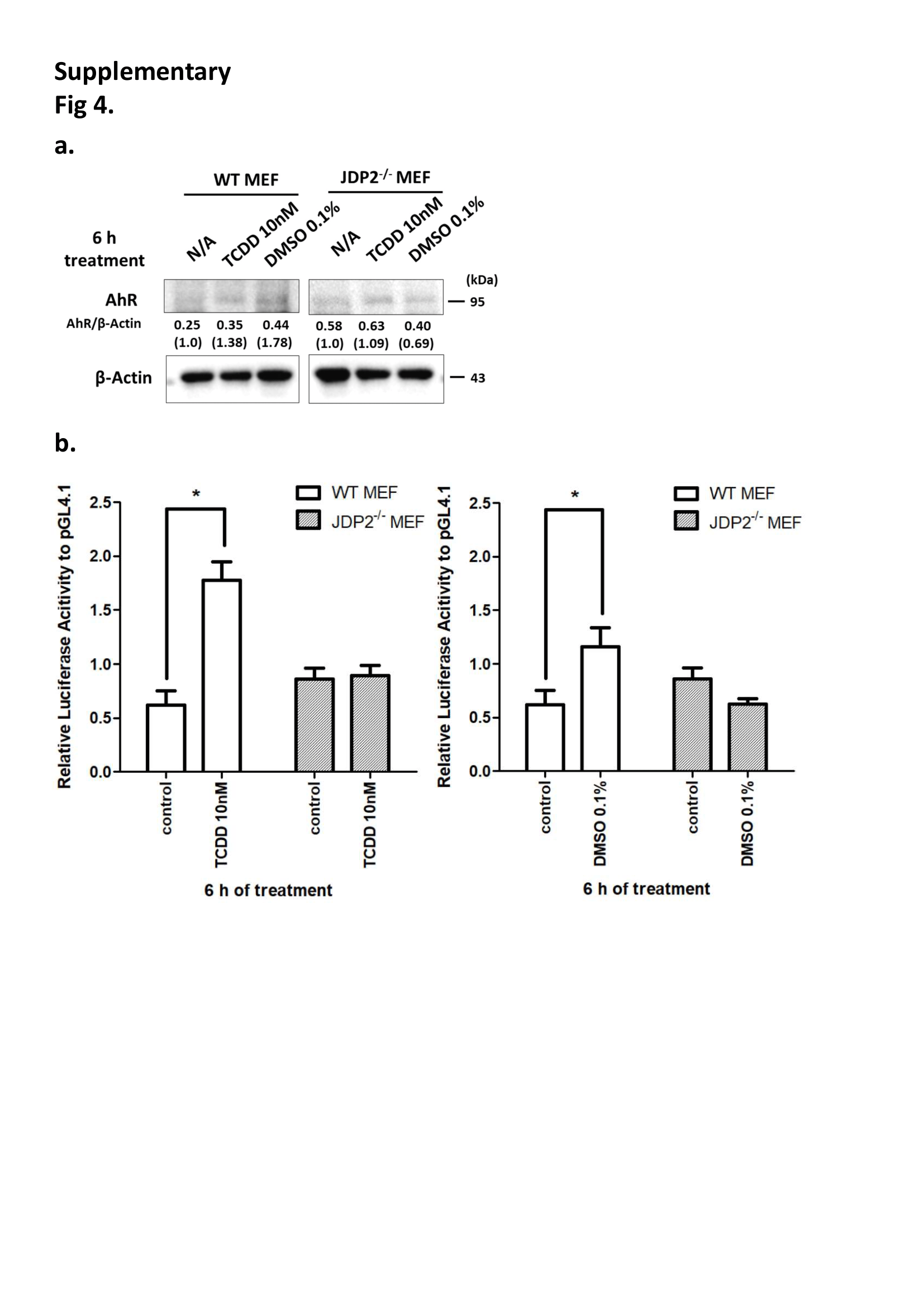
**

**
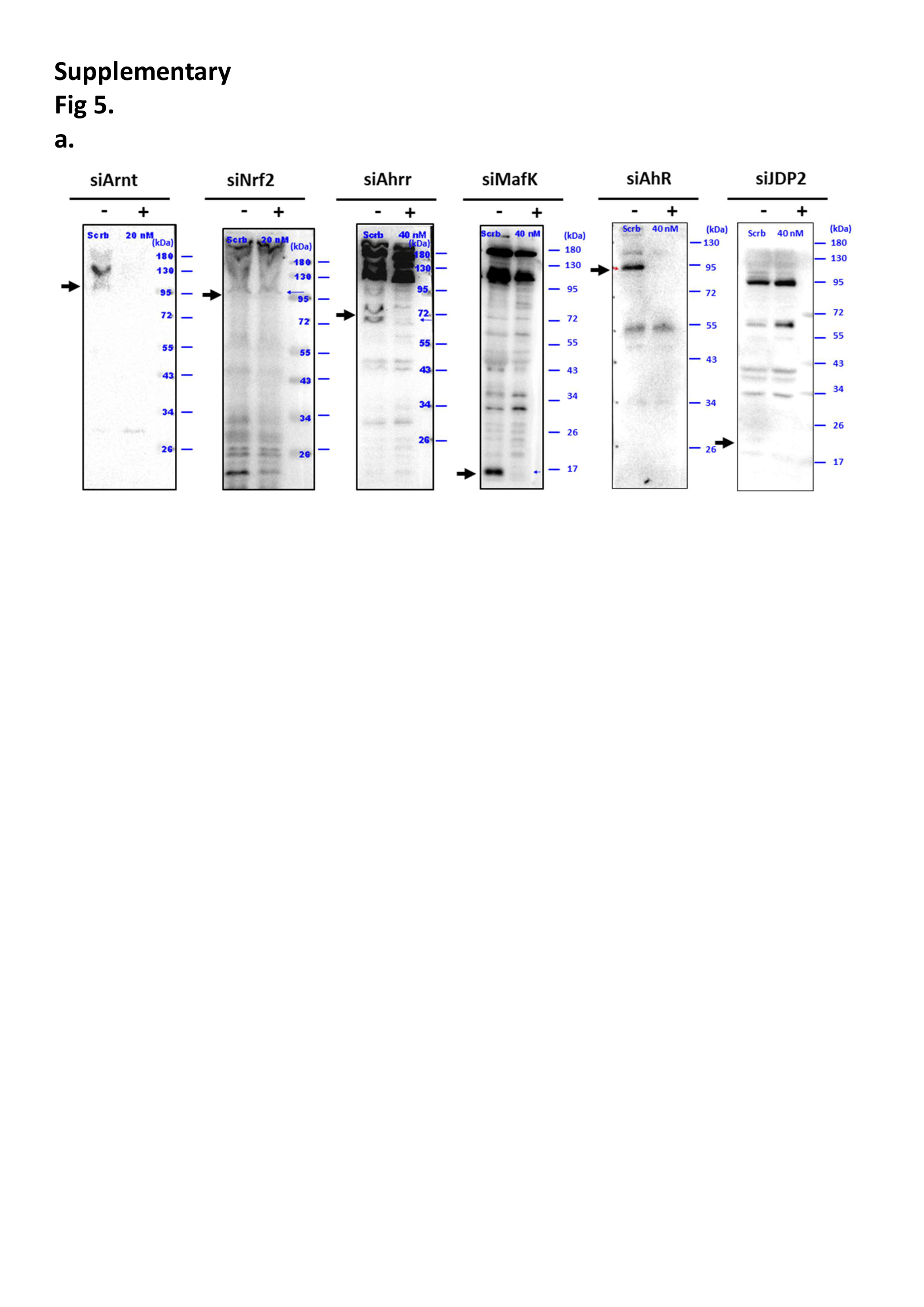
**

**
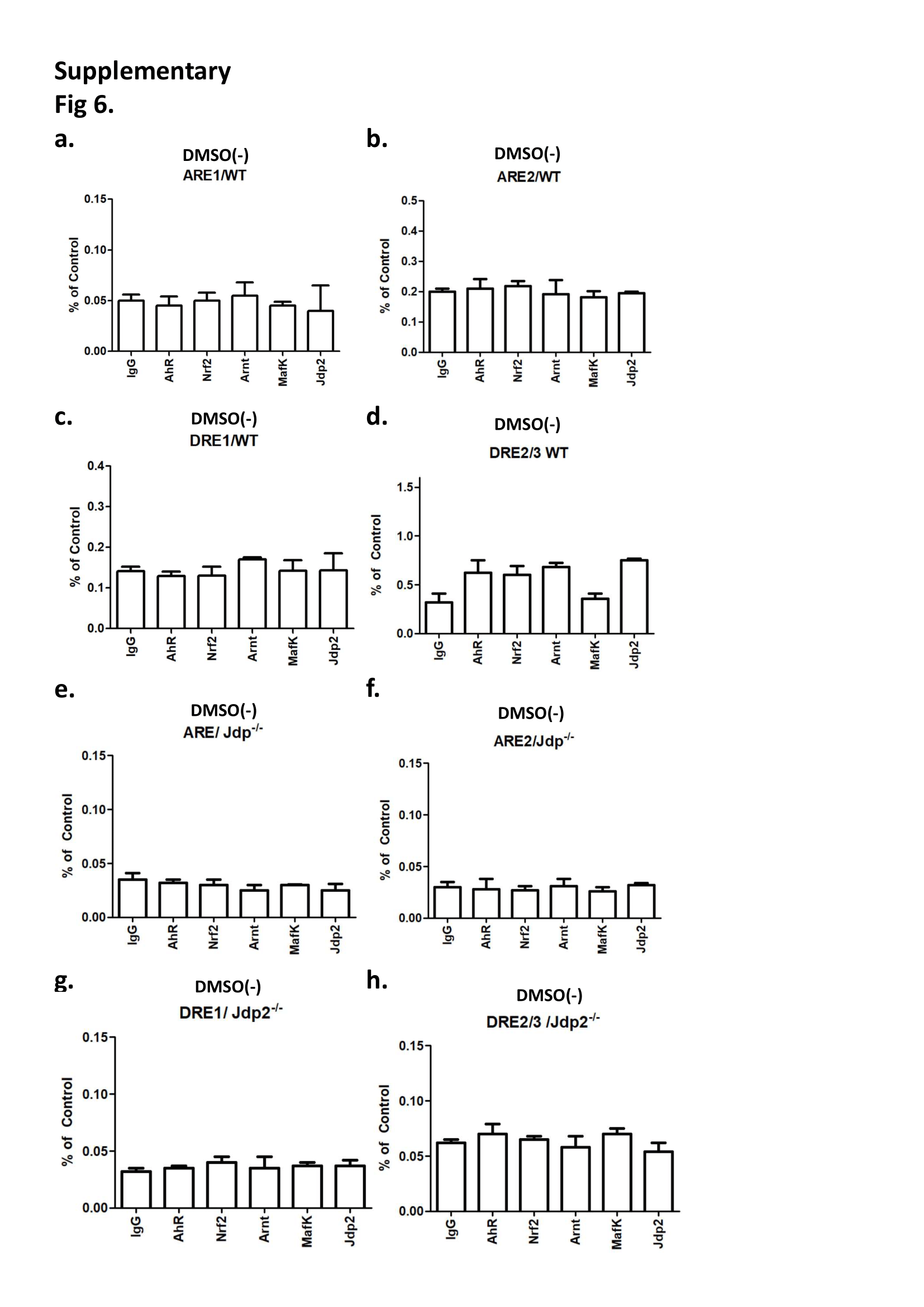
**

**
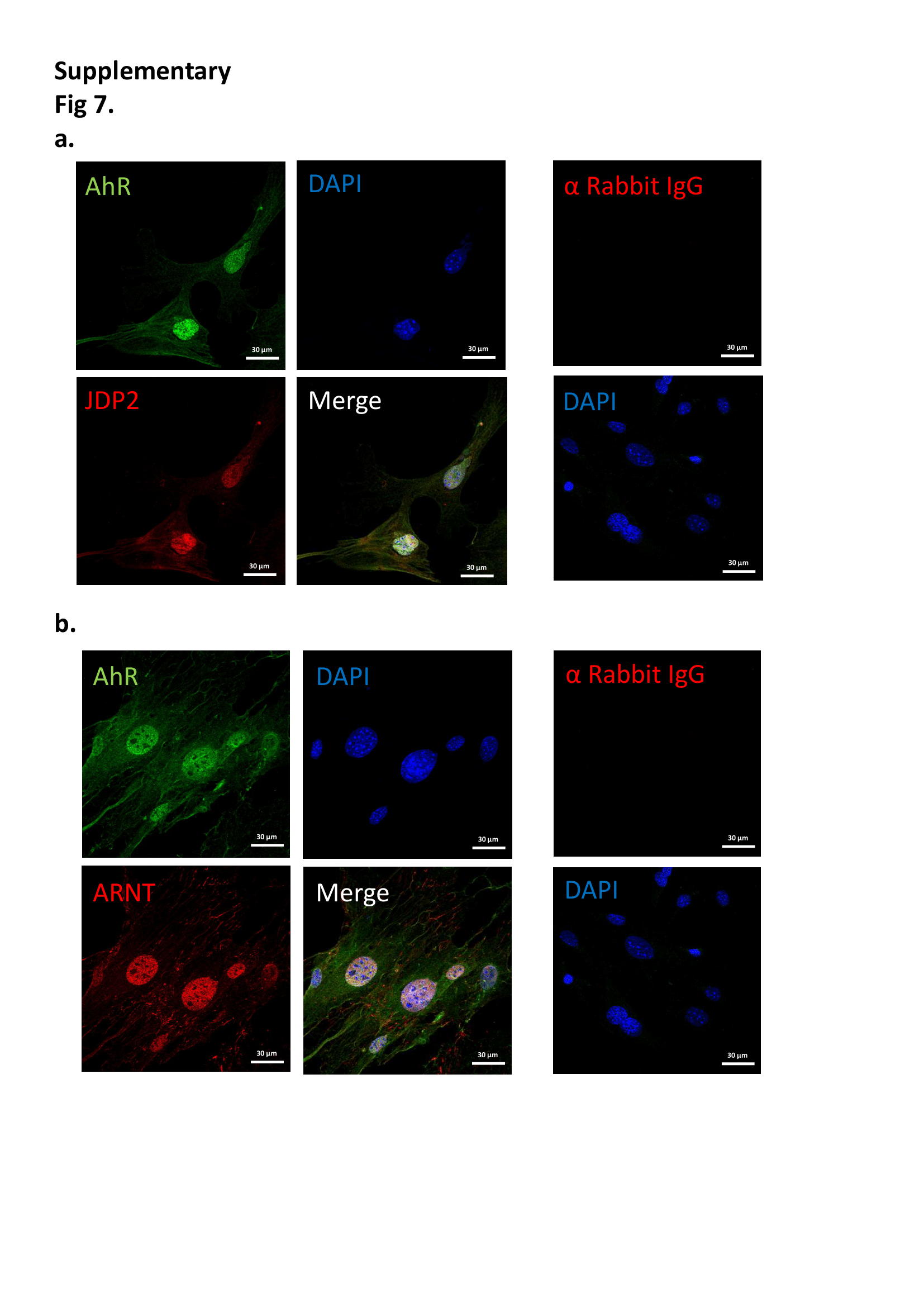
**

**
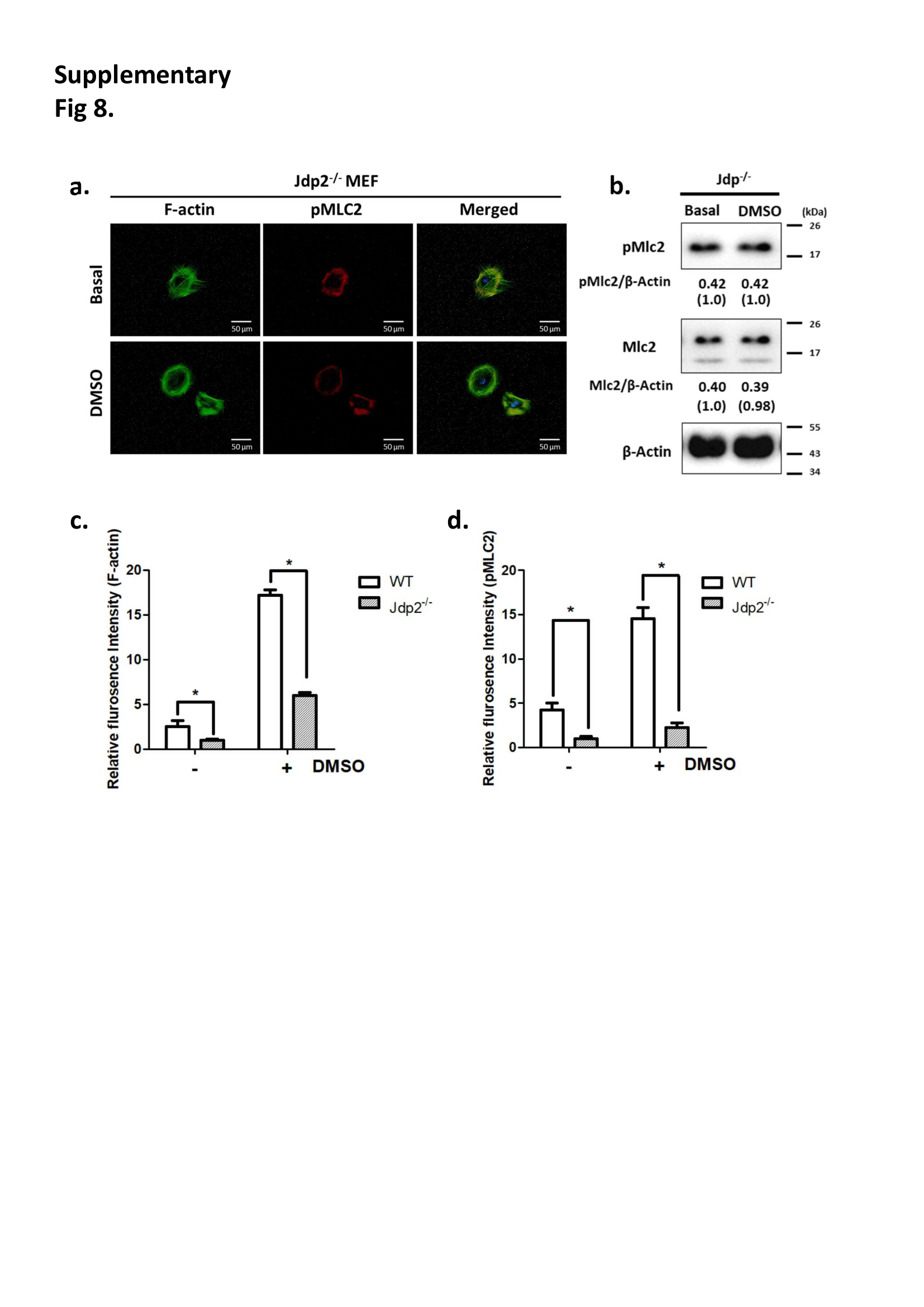
**

**
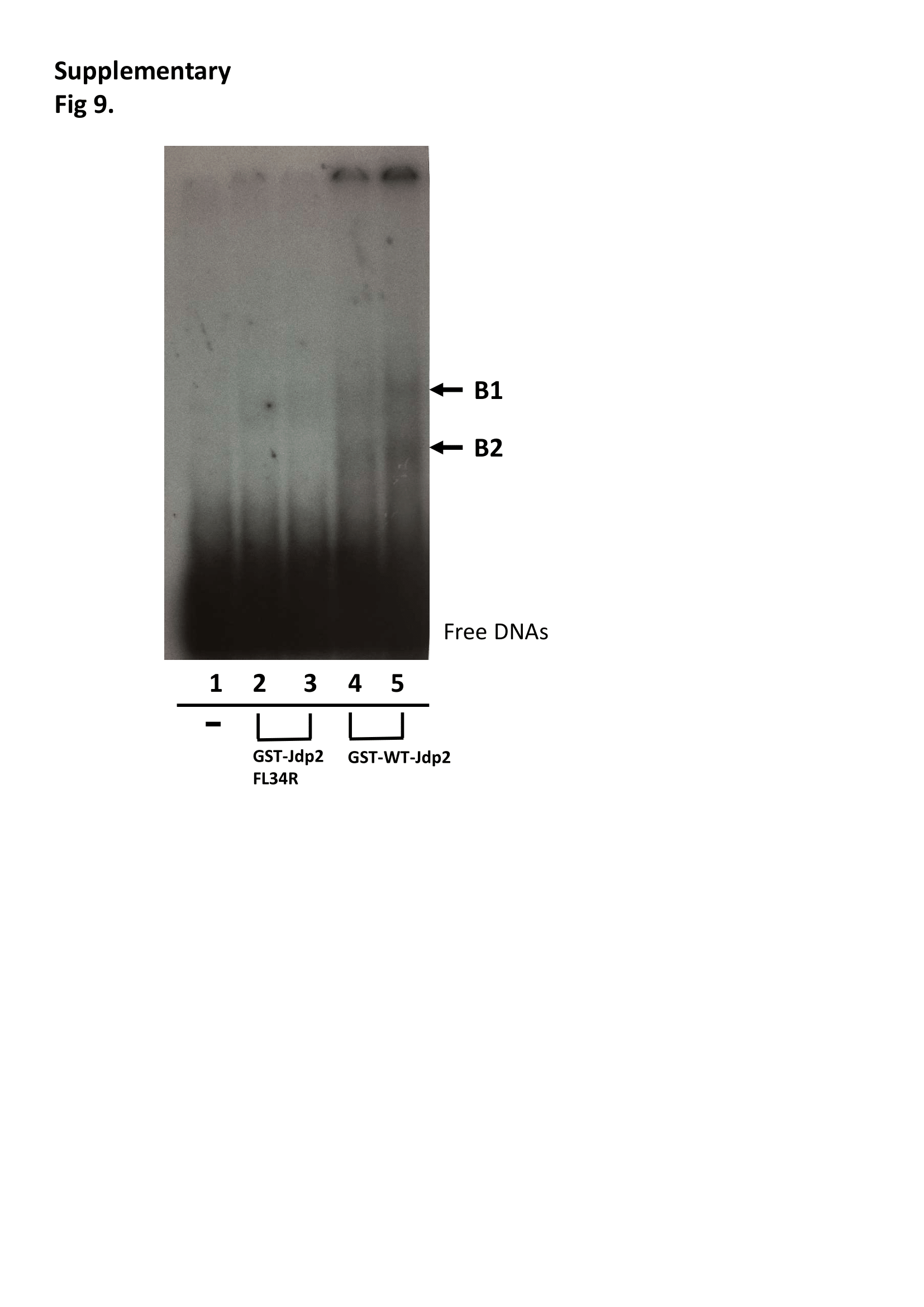
**

1. **Supplementary Tables**

**S Table1. Antibodies used in this study**

| **Antibody name** | **Company** | **Cat. No.** | **RRIDs** |
| --- | --- | --- | --- |
| Arnt | Cell Signaling Technology | CST#5537 | AB_10694232 |
| HIF-1beta | Gene Tex | GTX128795 | AB_2861418 |
| AhR | Santa Cruz Biotechnology | SC-8088 | AB_2223957 |
| AhR | Santa Cruz Biotechnology | SC-133088 | AB_2273721 |
| Nrf2 | Santa Cruz Biotechnology | SC-722 | AB_2108502 |
| Nrf2 | Gene Tex | GTX103322 | AB_1950993 |
| Mafk (NF-E2p18) | Santa Cruz Biotechnology | SC-477 | AB_2137821 |
| Jdp2 | A gift from Dr. A. Aronheim |  |  |
| Jdp2 | Santa Cruz Biotechnology | SC-517133 | AB_2861419 |
| Ahrr | Sigma-Aldrich | HpA019614 | AB_1855109 |
| β-actin | Santa Cruz Biotechnology | SC-47778 | AB_2714189 |
| FLAG-M2 | Merck Millipore | F1804 | AB_262044 |
| MLC2 | Cell Signaling Technology | CST#3672 | AB_10692513 |
| pMLC2 | Cell Signaling Technology | CST#3671 | AB_330248 |
| GAPDH | Millipore | MAB374 | AB_2107445 |
| Phalloidin (=F-actin) Alexa-Fluor488- Phalloidin | Thermo Fisher Scientific | A12379 | N/A |
| Normal Rabbit IgG | Cell Signaling Technology | CST#2729 | AB_1031062 |
| Normal Mouse IgG | Merck Millipore | 12-371 | AB_145840 |
| Anti-Rabbit IgG HRP | Cell Signaling Technology | CST#7074 | AB_2099233 |
| Anti-Mouse IgG HRP | Cell Signaling Technology | CST#7076 | AB_330924 |
| Anti-Goat IgG HRP | Santa Cruz Biotechnology | SC-2020 | AB_631728 |
| Alexa-Fluor ® 488 conjugated Goat anti-Mouse IgG | Thermo Fisher Scientific | A-11029 | AB_138404 |
| Alexa-Fluor® 488 conjugated Goat anti-Rabbit IgG | Thermo Fisher Scientific | A-11034 | AB_2576217 |
| Alexa-Fluor ® 594 conjugated Goat anti-Mouse IgG | Thermo Fisher Scientific | A-11032 | AB_2534091 |
| Alexa-Fluor ® 594 conjugated Goat anti-Rabbit IgG | Thermo Fisher Scientific | A-11037 | AB_2534095 |
| Annexin V | BD Bioscience | 51-65874X | AB_2888981 |

**S Table2. Primer sequence of Probes ARE1, ARE2, DRE1 and DRE2/3 in ChIP assay**

| **Regions** | **Primer sequences** | **Amplified size (bp)** |
| --- | --- | --- |
| ARE1 | Sense 5’-CCTGGTAAATCTTGATGTCTGGG-3’  Antisense 5’-ATGACGCAGGACGTAGTGAC-3’ | 159 |
| ARE2 | Sense 5’- CAGAATTTCCACCTTTTCCCACA-3’  Antisense 5’-AGGAAAGAACACAGGAGTGC-3’ | 223 |
| DRE1 | Sensei 5’- ACTGCGCGGGGTCG-3’  Antisense 5’-GTCCACCAGTTCGTCCTCC-3’ | 139 |
| DRE2/3 | Sense 5’- GACGAACTGGTGGACGGA-3’  Antisense 5’- GGAGAAACCCGCACGCTA-3’ | 145 |

**S Table3. Mutation of ARE1, ARE2, DRE1, DRE2, and DRE3 in AhR promoter**

| **Site** | **Primer sequences for mutation cis-element** |
| --- | --- |
| ARE1 | 5’- GGGAGTCACTACGTCCTCTTCCGCACCGTGCTGCGAAGAGGGTG-3’ |
| ARE2 | 5’- TTCCACCTTTTCCCACAACAGTCCCTTCAAGAAAGATGGAACATC-3’ |
| DRE1 | 5’- GGACCGGGCGCGGCGCTACATCGGGTTTCTCCTC-3’ |
| DRE2 | 5’-CTCGGTGCCCCACTTCCACGGCGGAGAGGCTCAGC-3’ |
| DRE3 | 5’- CGCGGCGGGCGGCACGTACACTGCCACCTCCCTTTGACGCTC-3’ |
| AP-1 | 5’- CTTCCATCTGTTTTGTTCCCGTACACCAGAATTTCCACCTTTTC-3’ |

**S Table4. siRNAs used in this study**

| **siRNA** | **Santa Cruz Co.** | **Other company** |
| --- | --- | --- |
| Control siRNA | SC-44234 |  |
| Negative control | #01 siRNA | Ambion ®. Thermo Fisher Scientific |
| AhR | SC-29658 |  |
| AhRR | SC-140918 |  |
| Nrf2 | SC-37049 |  |
| Arnt | SC-29734 |  |
| Mafk | SC-38104 |  |
| Jdp2 | SC-38018 |  |
